# Validity of self-assessment tools for cardiovascular risk behaviors: A systematic review

**DOI:** 10.1016/j.ajpc.2025.101316

**Published:** 2025-10-07

**Authors:** Wilhelmina F. Goevaerts, Joyce M. Heutinck, Mayke M.C.J. Van Leunen, Wessel W. Nieuwenhuys, Lonneke A. Fruytier, Cyrille Herkert, Jos J. Kraal, Ilse A.G. Rongen, Willem J. Kop, Yuan Lu, Hareld M.C. Kemps, Rutger W.M. Brouwers

**Affiliations:** a Department of Industrial Design, Eindhoven University of Technology, Eindhoven, the Netherlands; b Department of Cardiology, Máxima Medical Center, Eindhoven, Veldhoven, the Netherlands; c Department of Medical BioSciences, Radboud University Medical Center, Nijmegen, the Netherlands; d Faculty of Industrial Design Engineering, Delft University of Technology, Delft, the Netherlands; e Department of Medical and Clinical Psychology, Center of Research on Psychological Disorders and Somatic Diseases (*CoRPS*), Tilburg University, Tilburg, the Netherlands

**Keywords:** Lifestyle behavior, Validity, Self-assessment, Cardiovascular risk, Prevention, Digital health

## Abstract

**Background:**

A healthy lifestyle is crucial in mitigating cardiovascular disease risk. Numerous tools for cardiovascular risk behaviors have been developed that people can use for self-assessment purposes. However, the validity of these tools is insufficiently understood in the context of self-assessment. This systematic review examines the validity of self-assessment tools for cardiovascular risk behaviors, including lack of physical activity (PA), tobacco smoking, excessive alcohol consumption, unhealthy diet, and chronic psychological stress.

**Methods:**

The PubMed, Ovid Embase, and the Cochrane Library databases were searched. Studies investigating the validity of tools in the context of self-assessment (i.e., without active involvement of a healthcare professional) were included. We investigated criterion validity (i.e., comparison to a gold standard), convergent validity (comparison to similar measures), face and content validity, and reliability.

**Results:**

Thirty-one unique articles reporting on 37 separate validation studies were included, which examined a total of 49 distinct self-assessment tools (with tools for PA (*n =* 40), nutritional intake (*n =* 7), psychological stress (*n =* 1), and multiple domains (*n =* 1)). No validation studies were found for self-assessment of tobacco smoking or alcohol consumption. All wearable PA intensity assessment–energy expenditure studies demonstrated weak validity, both in laboratory and free-living conditions. Criterion validity was examined for only two nutritional intake tools, showing weak to moderate validity. For psychological stress and tools measuring multiple domains, only convergent validity was examined.

**Discussion:**

Behavioral self-assessment tools are predominantly focused on PA and nutritional intake, with limited evidence for good validity. There is a pressing need for developing and validating comprehensive and accurate self-assessment tools.

## Graphical abstract

**Figure:**
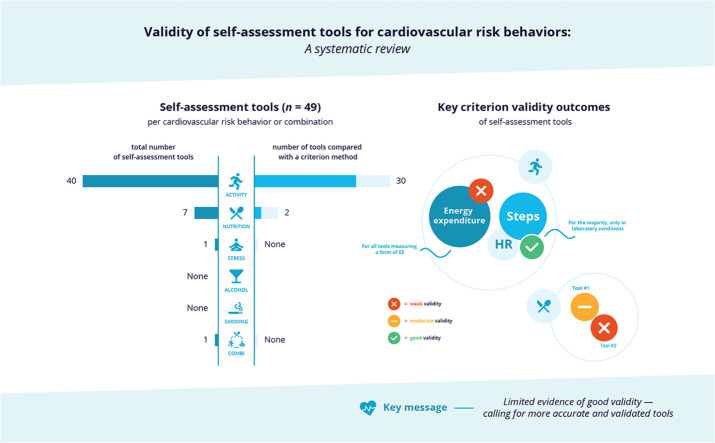

**Key Learning Points**

**What is already known**

• Cardiovascular diseases are the leading cause of global mortality, driven by traditional and lifestyle behavioral risk factors, with prevention focused on modifying behaviors to reduce risk.
• Self-assessment tools for cardiovascular risk behavior can empower individuals to recognize and modify cardiovascular risk behaviors, supporting lifestyle changes through increased awareness.
• The expanding use of self-monitoring tools for CVD prevention raises concerns about their validity, reliability, and comprehensiveness, necessitating further evaluation.

**What this study adds**

• The review reveals that most self-assessment tools for cardiovascular risk behaviors demonstrate weak to moderate validity, particularly in free-living conditions, with many relying on non-gold standard comparison methods.
• The review identifies gaps in self-assessment tools, especially for psychological stress, tobacco smoking, and alcohol consumption, suggesting a need for tools that address all cardiovascular risk behaviors comprehensively.
• The study emphasizes the need for further validation of self-assessment tools, which could eventually be integrated into clinical practice to enhance patient care, foster behavior change, and reduce cardiovascular disease risk.

Alt-text: Unlabelled box

## 1 Background

Cardiovascular diseases (CVDs) are the leading cause of death globally, responsible for 17.9 million deaths in 2019, representing 32% of all global deaths [1]. Traditional risk factors for CVD include hypertension, hypercholesterolemia, diabetes mellitus, and existing cardiovascular conditions such as prior heart disease or stroke. Often, before these risk factors become evident, individuals engage in behaviors that increase cardiovascular risk [2], such as physical inactivity, tobacco smoking, excessive alcohol consumption, unhealthy eating habits, and chronic psychological stress. Primary prevention, which focuses on altering these risk behaviors in otherwise healthy individuals, can reduce the development of traditional risk factors and ultimately decrease the morbidity and mortality associated with CVD.

Over the past few decades, numerous assessment tools for cardiovascular risk behaviors have been developed. These tools are widely used in epidemiological studies, intervention research, and clinical practice, such as in cardiac rehabilitation. However, they are not always suitable for use by the general public without professional guidance. Behavior change theories suggest that self-assessment of cardiovascular risk behaviors can enhance an individual’s awareness, empowerment, and, ultimately, drive behavior change [3]. Lifestyle assessment and tracking through self-assessment and self-monitoring tools therefore have the potential to initiate and support this change in the general public, thereby reducing CVD risk.

The market for consumer wearable activity trackers is expanding rapidly, providing many people opportunities to self-monitor physical activity (PA). The latest generation of these devices includes multiple sensors, which are allegedly comparable or even superior to advanced research instruments [4]. Additionally, various consumer mobile applications exist for monitoring other lifestyle domains, such as food diaries for tracking energy intake (EI), and wearable devices that assess sleep quality and daily stress levels. While self-monitoring is a critical component of behavior change, the effectiveness of these tools hinges on their comprehensiveness [5], accuracy, validity, and reliability—yet it remains uncertain whether sufficient evidence supports these aspects.

Given the anticipated role of digital self-assessment tools in the future of CVD prevention and rehabilitation, there is a pressing need to evaluate the current state and validity (i.e., screening accuracy) of these lifestyle self-assessment tools. Therefore, this systematic review aims to examine the validity of self-assessment tools for cardiovascular risk behaviors, including PA, tobacco smoking, alcohol consumption, nutritional intake, and psychological stress.

## 2 Methods

This systematic review is reported in accordance with the Preferred Reporting Items for Systematic Reviews and Meta-Analyses (PRISMA) criteria [6] (see Appendix A). The protocol for this systematic review was registered at the International Prospective Register for Systematic Reviews (PROSPERO), registration number CRD42017070945 [7].

### 2.1 Search strategy

The following electronic databases were searched: PubMed, Ovid Embase, and the Cochrane Library. Searches were conducted in June 2017 and updated in August 2023. The search strategy included combinations of the following: construct (PA, tobacco smoking, alcohol consumption, nutritional intake, and/or psychological stress) and the measurement properties (validation study, validation or validity, and self-evaluation, self-check, self-monitoring, or self-management). Searches were adapted to each database, alongside the use of appropriate Boolean operators and database-specific filters (see Appendix B). The authors (WG (lead), RB, and IR) worked with information specialists from the medical library of the Máxima Medical Centre (Veldhoven, The Netherlands) to refine the search strategies.

### 2.2 Eligibility criteria for included studies

Studies that evaluated the validity of self-assessment tools for cardiovascular risk behaviors were included. For this review, self-assessment tools for cardiovascular risk behaviors were defined as tools used to evaluate a person’s behavior in one of the following domains: PA, tobacco smoking, alcohol consumption, nutritional intake, and/or psychological stress. These tools may appear in different forms, e.g., questionnaires, diaries, mobile or web-based applications, or wearable devices. A self-assessment tool was defined as a tool used by the person whose behavior is being assessed (i.e., not by a healthcare professional (HCP)), and providing feedback to the person regarding their health behavior. A validity study was defined as a study comparing a self-assessment tool to another test which has already been validated, preferably the gold standard for assessment of behavior in the specified domain (e.g., for assessing the validity of a self-assessment PA questionnaire, the questionnaire can be compared to the doubly labeled water (DLW) method [8], which is considered a criterion method for assessing energy expenditure (EE)). For this review, no context was specified, as self-assessment of cardiovascular risk behavior may take place—and prove helpful—in multiple settings (i.e., at a general practitioner, hospital, physical therapist, or school), and is not restricted to a specific population. A complete overview of the exclusion criteria for title/abstract and full text screening is provided in Table A.

**Table A:** Exclusion criteria.

***Exclusion criteria***	
***i.***	Abstract not available
***ii.***	Publication not in English
***iii.***	Publication type: Comment, editorial, letter, news, congress, or protocol
***iv.***	Publication type: Systematic review or meta-analysis
***v.***	Publication type: No empirical study or review of empirical studies
***vi.***	Population: Children or adolescents
***vii.***	Population: Animal study or studies
***viii.***	Intervention: No cardiovascular risk behavior, i.e., physical inactivity, tobacco use, excessive alcohol consumption, unhealthy diet, or (chronic) psychological stress **Definition per cardiovascular risk behavior in this review** • Physical fitness and physical activity: Evaluating levels of physical fitness, physical activity, exercise habits, and sedentary behavior • Tobacco smoking: Assessing smoking status and tobacco use • Alcohol consumption: Evaluating patterns of alcohol consumption • Nutritional intake: Assessing dietary patterns (e.g., total nutrition, or intake of fruits, vegetables, saturated fats, sodium, and other nutrients) • Psychological stress: Assessing psychological stress levels (i.e., as a response to chronic exposure to continuing (i.e., daily life) stressors)
***ix.***	Intervention: No self-assessment: cannot be filled in by patient/person individually (so without support or help from a professional) **Definition of (no) self-assessment in this review** • No self-report: Cannot be filled in or used by patient/person individually (so without support or help from a professional) • No self-evaluation: Is not used by the patient to evaluate/monitor their own behaviors (no *active* feedback is received)
***x.***	Intervention: Evaluation of medication
***xi.***	Outcome: No validation study
***xii.***	Outcome: No index- and reference-test described
***xiii.***	No full text available

**Table legend:**

This table summarizes the criteria used to exclude studies during the screening process.

In this review, the following measurement properties were examined: criterion validity, convergent validity, face validity, and content validity. Reliability data were also collected as it underpins research and practice in this area. There remains some debate over the terminology used for validity in the field of lifestyle behavior, particularly in relation to criterion and convergent validity. For the purpose of this review, we developed a level of evidence scheme to distinguish between validity studies that used different comparison measurement tools. The scheme is an adapted version of the level of evidence scheme developed by Philips et al. [9]. Table B provides a full explanation of what was considered to constitute each level of evidence per lifestyle domain.

**Table B:** Level of evidence for validity studies included in this review.

**Level of evidence for validity**		**Criteria of comparisons (examples)**					
		**Measurement tool under study**	**Comparison tool/measure**				
			**Physical activity**			**Nutritional intake**	**Psychological stress**
			*Energy expenditure*	*Heart rate*	*Sedentary behavior and step count*	*Energy expenditure and energy intake*	*(Perceived) stress levels*
Level 1	Criterion validity	Any self-assessment measurement tool to determine physical activity, nutritional intake, and/or psychological stress	DLW and calorimetry (direct and indirect) [8,10]	Electrocardiography (ECG), HR chest straps (Polar-brand), and pulse oximetry [11,12]	Direct observation [13]	*Golden standard:* Weighed Food Records (WFR) in laboratory setting; or DLW [14]	*Considered golden standard:* Direct observation: interview by psychiatrist or diagnostic interview based on validated screening tools [15,16]
Level 2	Convergent validity: measurement tool compared with a measure which is considered to have relatively high validity (but not considered criterion(a))	Direct observation protocols (newly devised)	1) Device based measurement tools with known higher validity than the tool under study 2) or directly measured by HCP	1) Device based measurement tools with known higher validity than the tool under study 2) or directly measured by HCP	1) Device based measurement tools with known higher validity than the tool under study 2) or directly measured by HCP	1) A method evaluating short-term intake, including both 24-hour recall and WFR of less than 7 days by dietician [17] 2) measured with recovery or concentration biomarkers in short term	(b)
		Device based measurement tools: combined heart rate and accelerometer, heart rate monitor, accelerometer, pedometer					
		Proxy reported measurement tool: questionnaire/diaries					
Level 3	Convergent validity: measurement tool compared with a measure which is considered to have relatively low validity	Direct observation protocols (newly devised)	1) Device based measurement tools with equal or lower validity than the tool under study 2) or measured by HCP, but not directly	1) Device based measurement tools with equal or lower validity than the tool under study 2) or measured by HCP, but not directly	1) Device based measurement tools with equal or lower validity than the tool under study 2) or measured by HCP, but not directly	1) A method evaluating long-term intake, including more than 7 days of dietary collection by dietician 2) or measured with recovery or concentration biomarkers in long term	(b)
		Device based measurement tools: combined heart rate and accelerometer, heart rate monitor, accelerometer, pedometer					
		Proxy reported measurement tool: questionnaire/diaries					
Level 4	Convergent validity: two (or more) of the same type of measurement tools being compared where neither tool is considered to have known higher validity	Device based measurement tools: combined heart rate and accelerometer, heart rate monitor, accelerometer	Proxy reported measurement tools: questionnaire, diaries (used by patients themselves)				
		Proxy reported measurement tool: questionnaire, diaries					

**Table legend:**

Abbreviations: DLW = Doubly Labeled Water; ECG = Electrocardiography; HCP = Healthcare Professional; HR = Heart rate; WFR = Weighed Food Records.

(a) In this review we do not consider research-grade devices as criterion for free-living validation studies since insufficient validity has been shown for these devices to be considered an alternative gold standard [13].

(b) The field of (digital) mental health tools is in its early stages [16]. Therefore, high-quality evidence of accuracy of measurement tools other than the currently considered gold standard and self-report tools is lacking. Hence, no convergent level 2 and convergent level 3 are described for measuring psychological stress. In this review, studies using methods other than the criterion method or proxy-reported tools, such as concentration biomarkers, will be assessed for convergent validity in the same way as studies measuring nutritional intake.

### 2.3 Screening for relevant studies to include in the review

Following the searches, all identified articles were imported into a reference management program (Endnote X7) and duplicates were removed using the Bramer method [18]. Titles and abstracts from the search in 2017 were screened for inclusion by the authors RB and IR. Titles and abstracts from the search in 2023 were screened for inclusion by the lead review author (WG). Full texts of all potentially relevant studies from both searches were double-screened by two reviewers working in pairs (WG, WN, HK, JK, LF, and CH), with each reviewer screening the texts independently.

### 2.4 Data extraction of individual studies included in the review

Data from all relevant studies were extracted by two reviewers working in pairs (WG, WN, HK, JK, LF, and CH), with each reviewer extracting the data independently using a pre-piloted data extraction form. Any discrepancies were resolved by discussion within the pairs. Extracted information included: study characteristics, participant characteristics, details of the measurement tool explored (e.g., kind of activity tracker (e.g., multisensory (wearable) device, accelerometer-only (wearable) device, pedometer, or other) or digital tool (e.g., platforms (systems combining mobile applications and websites), mobile applications, or websites)), details of the reference methods used for comparison, lifestyle domain (i.e., PA, tobacco smoking, alcohol consumption, nutritional intake, psychological stress, or a combination of lifestyle domains), measurement properties assessed (i.e., criterion, convergent, content, and/or face validity), the results of the study, reliability evidence, and risk of bias assessment.

Secondary analyses explored type of measurement (telemonitoring (i.e., continuous or remote monitoring over time), repeated measurements (i.e., multiple assessments taken at regular intervals) and their frequency, or one-off measurement (i.e., a single isolated assessment conducted at a specific point in time), the availability of the measurement tools, the device placement (for activity trackers), the input method/interactivity (e.g., what kind of input type for digital food diaries) and (sensor) technology used (e.g., bi-axial or triaxial accelerometer).

### 2.5 Risk of bias assessment

The risk of bias of the included studies was assessed by two reviewers working in pairs (WG, WN, HK, JK, LF, and CH) using 11 items derived from the QUADAS-2 [19] (Quality Assessment of Diagnostic Accuracy Studies-2) tool. The following characteristics were considered: spectrum of participants or patients, reference standard, delay between tests, partial verification, differential verification, incorporation, test and diagnostic review bias, clinical review, uninterpretable results, and withdrawals. Any discrepancies were resolved by discussion or, if necessary, by a third reviewer. Results are presented in a narrative summary.

### 2.6 Syntheses

Studies commonly use a number of different statistical analyses. In order to demonstrate consistency in the interpretation of the results across studies we scoped the relevant literature for guidance. We found no consensus in the literature as to which statistical test results indicate weak, moderate, or good validity. However, in line with a number of previous reviews [9,11,20,21] of this type we defined acceptable limits of percentage difference and provide indications of weak, moderate, and good correlation ranges.

Our interpretation of measurement accuracy was focused on acceptable limits of percentage difference of ±3% in controlled settings and a percentage difference of ±10% in semi-free- and free-living settings [13], as outlined in previous work [11,[21], [22], [23], [24]]. We interpreted correlation coefficients (Pearson’s and Spearman’s) as follows: 0 to <0.6, weak; ≥0.6 to <0.8, moderate; ≥0.8 to 1.0, good correlation, in line with previous reviews of this type [9,20,25,26].

Since studies used different methods of analysis and reporting of reliability (e.g., inter- and intra-device reliability for trackers [11], or reproducibility for food diaries [27]), reliability results are presented in a narrative summary.

We summarized the validity results of the studies which aimed to compare a particular measurement property (separated by lifestyle parameter) of a particular measurement tool (e.g., Apple Watch). We have included summaries of this information in Tables D, E, and F. If a study described a statistical interpretation other than a percentage difference or correlation, the authors’ interpretation is presented in the summaries.

## 3 Results

### 3.1 Study selection

**Fig. 1:**
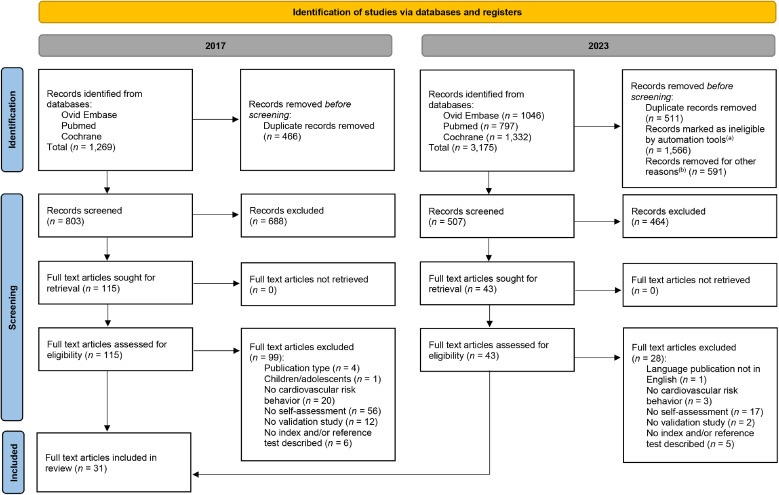
PRISMA flow chart of included studies. **Figure legend:** The flow diagram illustrates the selection process for studies included in the systematic review. ^(a)^ Conference abstracts (Ovid Embase) and trial registrations (Cochrane) were removed via automation tools. ^(b)^ Already screened records from the search of 2017 were removed.

A total of 3,175 records were identified from the databases. After duplicate records, conference abstracts (Ovid Embase), and trial registrations (Cochrane Library) were removed via automation tools, a total of 1,310 records remained (803 from the initial search (2017), and 507 from the updated search (2023)). After title and abstract screening, 158 articles were included for full text screening, and 31 articles were included in the review (Fig. 1). Sixteen articles were retrieved following the initial search and a further fifteen from the updated search. A list of excluded studies with reasons can be found in Appendix C.

### 3.2 Description of studies

An overview of information on all included articles is presented in Table C. The articles were published from 2009 to 2021. From the 31 articles, 22 reported on PA, none on tobacco smoking, none on alcohol consumption, seven on nutritional intake, one on psychological stress, and one on a combination of lifestyle domains (PA, nutritional intake, and psychological stress). The majority of the articles conducted their studies in high-income countries: United States of America (USA)(*n =* 7), Australia (*n =* 5), Canada (*n =* 3), The Netherlands (*n =* 3), Japan (*n =* 3), Denmark (*n =* 2), Germany (*n =* 2), United Kingdom (UK)(*n =* 2), Belgium (*n =* 1), Finland (*n =* 1), Spain (*n =* 1), and Sweden (*n =* 1). One study was conducted in Indonesia, which is considered an upper- to middle-income country. None of the studies were conducted in low-income countries. The majority of the articles reported that funding was received for their research (*n =* 20)(see Appendix D for funding sources of studies). Twenty-nine articles reported gender and the majority of the articles included both male and female participants in their studies: four articles included only females and one article included only male participants. 28 articles reported age, with total mean age ranging from 22.0 to 69.9 years. The majority of the articles (*n =* 19) included only healthy participants, two articles included a combination of healthy participants and participants suffering from obesity, two articles included patients with chronic obstructive pulmonary disease, two articles included participants with type 2 diabetes mellitus, one article reported on participants with CVD, one article included patients with non-specific low back pain, and one article included patients with several diseases or comorbidities. Five articles investigated reliability in their own form: inter-device reliability (*n =* 1), intra-device reliability (*n =* 1), test-retest reliability (*n =* 1), reproducibility (*n =* 1), and inter-method reliability (*n =* 1).

**Table C:** Summary of included studies validating self-assessment tools for cardiovascular risk behaviors.

**Study author(s), year, location, reference**	**Device names**	**Device type (technology; placement)**	**No. of patients included (analyzed)**	**Mean age (SD)**	**Percent male (n)**	**Population**	**Lifestyle parameter**	**Free-living/laboratory; study period**	**Reference test**	**Validity type**	**Measurement kind; frequency; input method**
**Studies evaluating self-assessment tools assessing PA (*n* = 22)**											
Åkerberg et al., 2016, Sweden, [28]	Yamax LS2000	Pedometer (spring-suspended lever-arm technology; waist)	20 (20)	40 (NA)	45% (9)	Healthy	PA; step count	Laboratory; tests in six environments in one day	Direct observation	Criterion (1)	Telemonitoring
	Pedometer FREE GPS+	Mobile application (iOS – 3-axis ACC)									
Chowdhury et al., 2017, UK, [4]	Apple Watch (Series 1)	Wearable multisensory device (3-axis ACC, PPG, and Gyro; wrist)	1. 30 (30) 2. 30 (30) Total: 30 (30)(same sample)	1. 27 (6) 2. 27 (6)	1. 50% (5) 2. 50% (5)	Healthy	PA; EE	1. Semi-free-living 2. Free-living (36 hours minimum)	1. Indirect calorimetry 2. Research-grade devices (Actiheart and BodyMedia Core)	1. Criterion (1) 2. Convergent (2)	Telemonitoring
	Microsoft Band	Wearable multisensory device (3-axis ACC, PPG, Gyro, barometer, and GPS; wrist)									
	Fitbit Charge HR	Wearable multisensory device (3-axis ACC, PPG, and Alt; wrist)									
	Jawbone UP24	Wearable accelerometer (3-axis; wrist)									
Dooley et al., 2017, USA, [29]	Apple Watch (Series 1)	Wearable multisensory device (3-axis ACC, PPG, and Gyro; wrist)	62 (62)	22.55 (4.34)	41.94% (26)	Healthy	PA; HR and EE	Laboratory: Exercise protocol on treadmill	Indirect calorimetry and HR chest strap	Criterion (1)	Telemonitoring
	Fitbit Charge HR	Wearable multisensory device (3-axis ACC, PPG, and Alt; wrist)									
	Garmin Forerunner 225	Wearable multisensory device (3-axis ACC, PPG, and GPS; wrist)									
Gomersall et al., 2016, Australia, [30]	Fitbit One	Wearable multisensory device (3-axis ACC and Alt; hip)	32 (29)(c)	39.6 (11)	10.34% (3)	Healthy	PA; step count, MVPA, and SB	Free-living; Two occasions, 7-days each	Research-grade accelerometer (ActiGraph GT3X+)	Convergent (2)	Telemonitoring
	Jawbone UP	Wearable accelerometer (3-axis; wrist)									
Holbrook et al., 2009, USA, [31]	Omron Healthcare HJ-151	Pedometer (single piezoelectric sensor; waist)	1. 34 (34) 2. 31 (31) Total: 47 (NA)	24 (4.4)(d)	51.06% (24)(d)	Healthy	PA; step count	Laboratory; a. 100-m walking b. 1-mile self-paced walking	Direct observation	Criterion (1)	Telemonitoring
	Omron Healthcare HJ-720ITC	Pedometer (dual piezoelectric sensor; waist)									
Leth et al., 2017, Denmark, [32]	Garmin Vívofit 2	Wearable accelerometer (3-axis; wrist)	1. 22 (22) 2. 9 (9) Total: 31 (31)	1. 31.1 (8.03) 2. 26 (5.1) Total: NA (NA)	1. 50% (11) 2. 44.4%(4) Total: NA (NA)	Healthy	PA; step count and HR	1. Laboratory: 100-m walking 2. Free-living: daily life (one night)	1. Direct observation: Gyro (Shimmer 3) 2. ECG (Shimmer 3)	1. Criterion (1) 2. Criterion (1)	Telemonitoring
	Fitbit Charge HR	Wearable multisensory device (3-axis ACC, PPG, and Alt; wrist)									
	Beddit Sleep Tracker	Sleep monitoring device (ballistocardiography); in bed)									
	Fitbit One	Wearable multisensory device (3-axis ACC and Alt; hip)									
	Fitbit Zip	Pedometer (3-axis ACC; waist or pocket)									
Middelweerd et al., 2017, The Netherlands, [33]	Fitbit One	Wearable multisensory device (3-axis ACC and Alt; hip)	34 (30)	23.9 (3.9)	33.33% (10)	Healthy	PA; step count, MPA, VPA, and MVPA	Free-living; 7 consecutive days	Research-grade accelerometer (ActiGraph GT3X+)	Convergent (2)	Telemonitoring
Orr et al., 2015, Canada, [34]	Accupedo	Mobile applications (iOS and Android – 3-axis ACC of phone)	1. 18 (18) 2. 11 (11) Total: 29 (29)	1. 28.78 (9.52) 2. 24.18 (3.06) Total: 27.07 (8.31)	NA (NA)	Healthy	PA; step count	1. Laboratory (4 conditions) 2. Free-living; three consecutive days	1. Direct observation 2. Research-grade pedometer (Yamax SW-200)	1. Criterion (1) 2. Convergent (2)	Telemonitoring
	Moves	Mobile applications (iOS and Android – 3-axis ACC and GPS of phone)									
	Runtastic	Mobile applications (iOS and Android – 3-axis ACC of phone)									
Powierza et al., 2017, USA, [35]	Fitbit Charge HR	Wearable multisensory device (3-axis ACC, PPG, and Alt; wrist)	22 (22)	22 (2)	45.45% (10)	Healthy	PA; HR	Laboratory; exercise protocol	ECG	Criterion (1)	Telemonitoring
Vooijs et al., 2014, The Netherlands, [36]	Fitbit Ultra	Wearable multisensory device (3-axis ACC and Alt; waist)	10 (9)	66.2 (4.4)	55.56% (5)	COPD	PA; EE	Free-living; 48 hours	Research-grade accelerometer (SenseWear Armband)	Convergent (2)	Telemonitoring
	Pam AM300	Wearable accelerometer (3-axis; waist)									
Boeselt et al., 2016, Germany, [37]	Polar A300	Wearable accelerometer (3-axis; wrist)	20 (20)	66.4 (7.4)	85% (17)	COPD	PA; EE, step count, TPA	Free-living; 3 consecutive days	Research-grade accelerometer (SenseWear Armband)	Convergent (2)	Telemonitoring
Ari Wibowo et al., 2020, Indonesia [38]	InaFit	Mobile applications (Android/GPS (distance) and camera with flashlight for HR)	35 (35)	NA (NA)	NA (NA)	T2DM	PA; HR and walking distance	Laboratory; walking test	Direct observation and pulse oximetry by HCP	Convergent (2)	One-off measurement: fitness test
Arrogi et al., 2018, Belgium, [39]	Axia Smart Active (ASA) Smart Chair (+ ASA mobile application)	Smart chair (pressure sensors in seat surface and backrest) (+ mobile application (iOS/Android))	1. 10 (10) 2. 10 (10) 3. 18 (18) Total: 28 (28)	1. 28 (5.6) 2. 28 (5.6) 3. 31 (8.3) Total: 30.0 (7.5)	1. 40.0 % (4) 2. 40.0 % (4) 3. 38.89% (7) Total: 60.71% (11)	Healthy	PA; SB	1. Laboratory: prescribed protocol 2. Free-living (2 hours) 3. Extended free-living (3 consecutive workdays)	1. Direct observation 2. Direct observation 3. Research-grade accelerometer (activPAL3c)	1. Criterion (1) 2. Criterion (1) 3. Convergent (2)	Telemonitoring
Bort-Roig et al., 2020, Spain, [40]	MetaWearC-sensor (+ Walk@Work mobile application)	Wearable accelerometer (3-axis; thigh) (with mobile application (iOS))	23 (20)	39.5 (8.1)	20% (4)	Healthy	PA; SB and LPA	Free-living; 3-8 consecutive working hours	Research-grade accelerometer (activPAL3m)	Convergent (2)	Telemonitoring
Ehrlich et al., 2021, USA, [41]	Fitbit Charge 3	Wearable multisensory device (3-axis ACC, Alt, and HR monitor; wrist)	15 (15)	27.0 (4.2)	0% (0)	T2DM (Gestational)	PA; step count	Laboratory: controlled tests (metronome assisted walking and stepping-in-place) (*n* = 15) and 200-m self-paced walk (*n* = 10)	Direct observation	Criterion (1)	Telemonitoring
Gill et al., 2018, UK, [42]	SitFIT	Wearable accelerometer (3-axis; trouser pocket)	21 (21)	NA (NA)	100% (21)	Healthy; Obesity	PA; step count and SB	Free-living; Up to 7 days (waking hours)	Research-grade accelerometer (activPAL^5^)	Convergent (2)	Telemonitoring
Heyken et al., 2021, Germany, [43]	Garmin Forerunner 35	Wearable multisensory device (3-axis-ACC, PPG, and GPS; wrist)	158 (158)	69.6 (NA)	65.82% (104)	CVD	PA; HR	Laboratory: Bicycle ergometer test	ECG	Criterion (1)	Telemonitoring
	Mio Fuse	Wearable multisensory device (3-axis ACC, PPG, and Gyro; wrist)									
	Fitbit Charge HR	Wearable multisensory device (3-axis ACC, PPG, and Alt; wrist)									
	Fitbit Surge	Wearable multisensory device (3-axis ACC, Gyro PPG, Alt, and GPS; wrist)									
	Withings Pulse Ox	Wearable multisensory device (3-axis ACC, optical HR sensor (fingertip use only), pulse oximeter sensor, and Alt; wrist)									
	Apple Watch (Series 1)	Wearable multisensory device (3-axis ACC, PPG, and Gyro; wrist)									
	Pearl FT FBT-50 V4	Wearable multisensory device (3-axis ACC, optical HR sensor, Gyro, and GPS; wrist)									
Jansson et al., 2022, Australia, [44]	*ecofit*	Mobile applications (iOS/Android)	1. 54 (54) 2. 13 (13) Total: 54 (54)(same sample)	1. 55.6 (14.2) 2. 45.6 (12.7) Total: 55.6 (14.2)	1. 33.33% (18) 2. 76.9 (10) Total: 33.33% (18)	Healthy	PA; Upper- and lower-body muscular fitness	Free-living: a. Sit-to-stand test b. Push-up test	Observation by HCP (at baseline, participants could do the test within 14-days of receiving the application)	Convergent (3)	One-off measurement; fitness test
Karinharju et al., 2021, Australia and Finland (50/50) [45]	Apple Watch (Series 1)	Wearable multisensory device (3-axis ACC, PPG, and Gyro; wrist)	26 (24)	42 (13)(a)	77% (20)(a)	Spina bifida, post-infectious autoimmune neuropathy (*n* = 3); epidural abscess (*n* = 2); transverse myelitis (*n* = 1); tumor (*n* = 1); cerebral palsy (*n* = 1); osteogenesis imperfecta (*n* = 1); motor neuron disease (*n* = 1)	PA; Wheelchair push counts	Semi-free-living; standardized protocol	Direct observation	Criterion (1)	Telemonitoring
Murakami et al., 2019, Japan, [46]	Fitbit Flex	Wearable accelerometer (3-axis; wrist)	1. 21 (19) 2. 21 (19) Total: 21 (19)(same sample)	1. 32.3 (9.6) 2. 32.3 (9.6)	1. 47.37% (9) 2. 47.37% (9)	Healthy	PA; EE	1. Semi-free-living; 1 standardized day 2. Free-living; 15 days	1. Metabolic chamber 2. DLW	1. Criterion (1) 2. Criterion (1)	Telemonitoring
	Jawbone UP24	Wearable accelerometer (3-axis; wrist)									
	Misfit Shine	Wearable accelerometer (3-axis; wrist)									
	Epson PULSENSE	Wearable multisensory device (3-axis ACC, PPG; wrist)									
	Garmin Vívofit 1	Wearable accelerometer (3-axis; wrist)									
	Tanita AM-160	Pedometer (3-axis ACC; pocket)									
	Omron CaloriScan HJA-401F	Wearable multisensory device (3-axis ACC, temperature sensor, weight measurement sensor; pocket)									
	Withings Pulse O2	Wearable multisensory device (3-axis ACC, PPG, and pulse oximeter; waist)									
Toledo et al., 2017, USA, [47]	BeWell24	Mobile applications (iOS/Android)	26 (17)	49.0 (8.9)(a)	85% (22)(a)	Healthy	PA; LPA and MVPA	Free-living; 11 weeks	Research-grade accelerometer (activPAL3c)	Convergent (2)	Repeated measurement; Dairy - reporting per day in 5-minute epochs
Zhuo et al., 2021, Canada, [48]	Garmin Vívofit 3	Wearable accelerometer (3-axis; wrist)	17 (17)	54.9 (11.7)	47.06% (8)	Healthy with non-specific low back pain	PA; step count, walking distance, and MVPA	Free-living; 12 weeks	Self-reported questionnaire (modified IPAQ-SF)	Convergent (4)	Telemonitoring
**Studies evaluating self-assessment tools assessing nutritional intake (*n* = 7)**											
Fukuo et al., 2009, Japan, [49]	Zaurus SL-C3000	PDA (Zaurus SL-C3000 - software name unknown)	60 (60)	52.8 (9.9)	55% (33)	Healthy; T2DM	Nutritional intake; EI, protein, fat, and carbohydrate	Free-living; 7/8 days consecutive measurements	24-hour recall interview by dietician	Convergent (2)	Repeated measurement; Record meal as soon as soon as possible after eating. PDA-based food diary – Food diary with pre-coded items and adjustable portion sizes (according to photographs)
Fuller et al., 2017, Australia, [50]	Boden Food Plate	Website (Computer)	76 (67)	42.3 (7.7)(a)	46% (35)(a)	Obesity	Nutritional intake; EI, protein, fat, and carbohydrate	Free-living; two times a three-day period (two working days and one weekend day) in a six-week study period	Written food diary by participants	Convergent (4)	Repeated measurement; Written diary completed during the day, at the end of the day this was used to complete the electronic diary. Food diary with pre-coded items and fixed portion sizes. Items could be virtually dragged onto the participant’s electronic plate
Goodman et al., 2015, Canada, [51]	Vitamin D Calculator	Mobile application (iOS)	50 (50)	22 (2)	50% (25)	Healthy	Nutritional intake; Vitamin D and calcium	Free-living; 3 non-consecutive days (two working days and one weekend day) over a 1-month study period	24-hour recall interview by researchers	Convergent (2)(e)	Repeated measurement; Daily: participants entered their vitamin D and calcium containing food intake items, intake of the items was based on a food composition database
Hutchesson et al., 2013, Australia, [52]	SP Health Weight Loss Platform	Website (Computer)	12 (9)	34.5 (11.3)	0% (0)	Obesity	Nutritional intake; EI	Free-living; 9-days consecutive measurements	DLW	Criterion (1)	Repeated measurement; Daily. Food diary with pre-coded items and adjustable portions size
McClung et al., 2009, USA, [53]	BalanceLog	PDA with ‘BalanceLog’-software	31 (26)(b)	23 (4)	92.31% (24)	Healthy	Nutritional intake; EI	Free-living; 7-days consecutive measurements	DLW	Criterion (1)	Repeated measurement; Daily. Food diary with pre-coded items and adjustable portion sizes; new items could be added
Matsuzaki et al., 2017, Japan, [54]	Asken	Website (Computer)	218 (163)	39.3 (10.3)	0% (0)	Healthy	Nutritional intake; EI, protein, fat, carbohydrate, calcium, sodium, iron, vitamin A, B1, B2, C, D, and E, cholesterol, and dietary fiber	Free-living; 7-days consecutive measurements	WFRs by participants	Convergent (4)	Repeated measurement; Daily. Food diary with pre-coded items: complete dishes with fixed ingredients and portion size
Ocké et al., 2021, The Netherlands, [55]	MijnEetmeter	Platform (Mobile to web; Android and iOS)	120 (100)	NA (NA)	35% (35); (64% female; 1% other)	Healthy	Nutritional intake; EI, protein, fat, saturated fatty acids, carbohydrate, mono- and disaccharides, and sodium	Free-living; 3 non-consecutive days (two working days and one weekend day)	24-hour recall interview by dietician	Convergent (2)	Repeated measurement; Daily; Food diary with pre-coded items and adjustable portion sizes, new foods can be added by user. Input method: text search and barcode scanning
**Studies evaluating self-assessment tools assessing psychological stress (*n* = 1)**											
Þórarinsdóttir et al., 2019, Denmark, [56]	Monsenso	Mobile application (Android)	46 (40)	35.24 (12.79)	45% (18)	Healthy	Psychological stress; perceived stress	Free-living; 4-month study period	Self-reported questionnaire (PSS)	Convergent (4)	Repeated measurement; daily self-assessment: Prompt by application once per day (8pm)
**Studies evaluating self-assessment tools assessing multiple lifestyle domains (*n* = 1)**											
Swendeman et al., 2018, USA, [57]	Ohmage	Platform (Mobile to web - open source/Android)	42 (29)	30.9 (6.3)	0% (0)	Healthy; Obesity	Combined: PA; LPA, MPA, and VPA. Nutritional intake; diet quality score Psychological stress; self-perceived stress	Free-living; 6-month study period	Self-reported questionnaire (FFQ, PSM-9), anthropometric bio measures, and bloodspot biomarkers	Convergent (4)	Repeated measurement; EMA (3 times per day for stress and diet) and diary (once per day for stress, diet, and PA)

**Table legend:**

Abbreviations for sensors: ACC = Accelerometer; Alt = Altimeter; Gyro = Gyroscope; GPS = Global Positioning System; PPG = Photoplethysmography.

Abbreviations for patient groups: COPD = Chronic Obstructive Pulmonary Disease; CVD = Cardiovascular disease; T2DM = Diabetes Mellitus Type 2.

Abbreviations for reference methods: DLW = Doubly Labeled Water; ECG = Electrocardiography; FFQ = Food Frequency Questionnaire; IPAQ-SF = International Physical Activity Questionnaire (Short Form); PSM-9 = Psychological Stress Measure; PSS = Perceived Stress Scale; WFR = Weighed Food Records.

(a) Calculated based on total included patients instead of total analyzed patients.

(b) Half of the volunteers were randomized to use the PDA or the written records.

(c) These were split into two groups: a group to validate the Fitbit One (*n* = 14) and a group to validate the Jawbone UP (*n* = 15).

(d) Based on the total number of analyzed participants (*n* = 47), experiments were done in subsets.

(e) The 24-hour dietary recall was performed by a research assistant instead of a dietician.

Within the 31 articles, 37 separate validation studies were identified, which examined the validity of 49 independent self-assessment tools. Overall, study sample size (included participants) ranged from nine to 218 and analyzed participants sample size ranged from nine to 163. The validation studies commonly assessed the measurement tools in free-living conditions (*n =* 24), used structured protocols that were reflective of daily living ('semi-free-living', *n =* 3), or were conducted in laboratory settings (*n =* 10). A total of 17 validation studies (45.9%) examined criterion validity, 14 studies (37.8%) examined convergent level 2 validity, one study (2.7%) examined convergent level 3 validity, and five studies (13.5%) examined convergent level 4 validity (see Table B for definitions). None of the studies examined content or face validity.

#### 3.2.1 PA self-assessment tools

40 self-assessment tools for PA specifically were assessed, from which 15 multisensory wearable devices, 11 wearable accelerometer-only devices, five pedometers, seven mobile applications, one sleep monitoring device, and one smart chair. Most of the tools were telemonitoring tools, except for two mobile applications that were used for one-off fitness tests, and one mobile application using daily repeated self-report measurements for collecting data on time spent in sedentary behavior (SB), light-intensity PA (LPA), and moderate-to-vigorous PA (MVPA). Study sample size (total analyzed participants) ranged from nine to 158. The assessed units of measure were: EE (kcal/min, kcal/day, metabolic equivalents (METs), and PA levels (PAL)) for 15 measurement tools, step count (steps/day) for 16 measurement tools, heart rate (HR) in beats per minute (bpm) for ten measurement tools, LPA (min/day) for two measurement tools, moderate-intensity PA (MPA)(min/day) for one measurement tool, MVPA (min/day) for four measurement tools, vigorous-intensity PA (VPA)(min/day) for one measurement tool, total PA (TPA)(min/day) for one measurement tool, SB (total sitting time; min/day) for five measurement tools, upper- and lower-body strength (number repetitions) for one measurement tool, walking distance (meters) for two measurement tools, and wheelchair push counts for one measurement tool. The most researched devices were the Fitbit Charge HR (*n =* 5), Apple Watch (*n =* 4), and Fitbit One (*n =* 3).

#### 3.2.2 Nutrition self-assessment tools

Seven self-assessment tools for nutritional intake specifically were assessed, from which three websites, two personal digital assistants (PDAs), one mobile application, and one platform. All measurement tools used daily repeated self-report measurements to collect nutritional intake data. Study sample size ranged from nine to 163 analyzed participants. The assessed units of measure were: EI (kJ/day and kcal/day) for six measurement tools, and micronutrients (carbohydrates, proteins, and fats, in g/day) for four measurement tools. Calcium (mg/day) and sodium (mg/day) were the only micronutrients assessed by two tools, whereas other micronutrients and food groups were only assessed by single tools.

#### 3.2.3 Psychological stress self-assessment tools

One measurement tool, a mobile application (Monsenso), was used to measure psychological stress specifically, using daily repeated self-report measurements (once per day). Study sample size (total analyzed participants) was 40. The assessed unit of measure was a self-perceived stress score (score 0 (no stress) - 2 (much stress)).

#### 3.2.4 Self-assessment tools measuring multiple lifestyle domains

One measurement tool assessed a combination of lifestyle domains (PA, nutritional intake, and psychological stress), which was a platform (Ohmage). The platform used daily repeated self-report measurements in the form of mobile Ecological Momentary Assessments (mEMAs), which prompted the user three times per day. Study sample size was 42. The assessed units of measure were: minutes spent in LPA, MPA, and VPA for PA; perceived meal quality (1 to 3) and perceived meal healthiness scores (1 to 5) for nutritional intake; and a self-perceived stress score (Likert scale 1 (not at all stressed) to 4 (very stressed)) for psychological stress.

### 3.3 Risk of bias

**Fig. 2:**
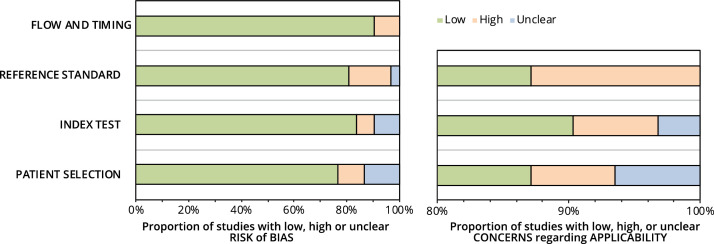
Graphical display of QUADAS-2 results. **Figure legend:** This figure presents the graphical summary of QUADAS-2 assessments across the included studies. Each bar represents a specific domain of risk of bias (i.e., Flow and Timing, Reference Standard, Index Test, Patient Selection) and applicability concerns. Green indicates low risk or concern, red denotes high risk or concern, and blue represents unclear risk or concern. The percentages on the x-axis indicate the proportion of studies within each category.

Risk of bias results are summarized in Fig. 2. The majority of the studies (>70%) had a low risk of bias in all QUADAS-2 domains. While the majority (60%) of studies had a low risk of bias related to the reference standard of measures, ∼15% had a mismatch between the examined outcome and the used reference test, which in this review was considered the most critical form of bias. Individual study risk of bias results are presented in Appendix E.

### 3.4 Summary of measurement properties of measurement tools, separated per lifestyle domain

The results will be discussed per lifestyle domain in line with the level of evidence scheme displayed in Table B. These results will be followed by the reliability results.

#### 3.4.1 Validity of tools measuring PA

In total, 22 articles with 28 validation studies examined the validity of self-assessment tools for PA. The majority of the studies examining tools measuring PA took place in free-living setting and semi-free-living setting (*n =* 18 (64.3%), free-living: *n =* 15, semi-free-living *n =* 3). Another ten studies (35.7%) were executed in laboratory settings. Criterion (*n =* 15), convergent level 2 (*n =* 11), convergent level 3 (*n =* 1), and convergent level 4 (*n =* 1) methods were used as comparison methods in these studies. We examined criterion validity for EE, step count, HR, and SB separately, followed by convergent level 2, 3, and 4 validity for tools measuring PA. Table D provides a summary of self-assessment tools that measure PA and their validity evidence.

**Table D:** Summary table of validity evidence of the self-assessment tools for measuring physical activity [4,[28], [29], [30], [31], [32], [33], [34], [35], [36], [37], [38], [39], [40], [41], [42], [43], [44], [45], [46], [47], [48],^57^].

*Criterion validity for EE*

Criterion validity for EE was examined in three studies, in which the validity of 12 activity trackers was investigated. Criterion validity was examined in both laboratory and free-living settings for two devices: the Fitbit Charge HR and the Apple Watch. Criterion validity was examined in semi-free-living or free-living setting only for nine devices: Jawbone UP24, Microsoft Band, Fitbit Flex, Misfit Shine, Epson PULSENSE, Garmin Vívofit 1, Tanita AM-160, Omron CaloriScan HJA-401, and the Withings Pulse O2. Criterion validity was examined in only laboratory setting for the Garmin Forerunner 225. All cases demonstrated weak validity.

*Criterion validity for step count and wheelchair pushes*

Six studies used the criterion method of direct observation for the outcomes step count (*n =* 5) and wheelchair pushes (*n =* 1). For 12 devices criterion validity was examined for step count, of which eight devices were only tested in laboratory conditions, and four were tested in both free-living and laboratory conditions. Only two measurement tools demonstrated good criterion validity in laboratory conditions, which were the Omron Healthcare HJ-151 pedometer and Omron Healthcare HJ-720ITC pedometer. Only the Fitbit One demonstrated good criterion validity in free-living conditions, but demonstrated weak criterion validity in laboratory conditions. All other devices showed weak validity in either free-living or laboratory conditions, except for the Garmin Vívofit 2, which showed moderate criterion validity in laboratory conditions. The validity of wheelchair push count was only examined in one study in semi-free-living conditions. The investigated activity tracker (Apple Watch) demonstrated weak validity.

*Criterion validity for HR*

Criterion validity for HR measurement tools was investigated in four studies: the criterion method of electrocardiography (ECG) for the outcome of HR was used in three studies and one study used the HR chest strap criterion method. Nine devices were examined for criterion validity, from which seven were tested in laboratory only conditions, one device was tested in both laboratory and free-living conditions (Fitbit Charge HR), and one device was tested in free-living conditions only (Beddit Sleep Tracker). Validity results were mixed. The Garmin Forerunner 35 and Mio Fuse demonstrated good validity in laboratory settings, and the Beddit Sleep Tracker demonstrated good validity in free-living setting. The Fitbit Charge HR showed contradictory results as it demonstrated weak validity twice and good validity once in laboratory conditions, and good validity in free-living conditions. Similarly, the Apple Watch showed both weak validity and strong validity in laboratory conditions. Two devices demonstrated moderate criterion validity (Fitbit Surge and Pearl FT FBT-50 V4) and two other devices showed weak criterion validity (Garmin Forerunner 225 and Withings Pulse Ox).

*Criterion validity for SB*

Criterion validity for SB was only examined for one smart chair with accompanied mobile application, which showed mixed validity results (i.e., weak in laboratory conditions and good in free-living conditions).

*Convergent level 2 of tools measuring PA*

All validation studies that used convergent level 2 validation methods (*n =* 11) used research-grade devices as comparison: ActiGraph GT3X+ (*n =* 2), Actiheart and BodyMediaCore (*n =* 1), Yamax SW-200 (*n =* 1), SenseWear Armband (*n =* 2), pulse oximeter (*n =* 1), and activPAL3c (*n =* 4). The only self-assessment tool that was tested in laboratory conditions was the InaFit mobile application, which showed good convergent validity for HR and walking distance. All studies examining the convergent level 2 validity of PA intensities (i.e., LPA, MPA, MVPA, VPA, and TPA) of measurement tools (Fitbit One, Polar A300, Jawbone UP, MetaWearC sensor (+ Walk@Work mobile application), and BeWell24 mobile application) demonstrated weak validity. The validity regarding EE was also examined for the Fitbit Ultra, Pam AM300, and Polar A300, which demonstrated moderate (Fitbit and Polar) and weak (Pam) validity.

*Convergent level 3 of tools measuring PA*

Only one study used a convergent level 3 method for validation and was also the only one to exame the outcomes of upper- and lower-body muscular fitnessassessed via the *ecofit* mobile application. The application showed moderate validity for lower-body muscular fitness and strong validity for upper-body muscular fitness.

*Convergent level 4 validity of tools measuring PA*

A convergent level 4 method (self-reported modified IPAQ-SF questionnaire) was used in only one study, which examined the validity of step count, MVPA, and walking distance of the Garmin Vívofit 3. Weak validity was demonstrated for all PA outcomes.

#### 3.4.2 Validity of tools measuring nutritional intake

All studies (*n =* 7) examining tools measuring nutritional intake only took place in free-living settings. Criterion (*n =* 2), convergent level 2 (*n =* 3), and convergent level 4 (*n =* 2) methods were used as comparison methods. Table E provides a summary of self-assessment tools that measure nutritional intake and their validity evidence.

**Table E:** Summary table of validity evidence of the self-assessment tools for measuring nutritional intake [[49], [50], [51], [52], [53], [54], [55],^57^].

*Criterion validity of tools measuring nutritional intake*

The criterion method of DLW for the outcome of EI only was used in two studies. The studies showed moderate (BalanceLog (PDA)) and weak (SP Health Weight Loss Platform (website)) validity results.

*Convergent validity of tools measuring nutritional intake*

Three studies used a convergent level 2 method (24-hour recall interview by dietician) for the outcomes of EI and nutrients with the majority of the evidence reporting moderate validity. Two other studies used convergent level 4 methods (written food diary by participant and WDR by participants) for the outcomes of EI and nutrients. High validity was shown for EI for two websites (the Boden Food Plate website and the Asken website), but showed mixed results for nutrients outcomes.

#### 3.4.3 Validity of tools measuring psychological stress

There was one study that only examined the validity of a measurement tool for psychological stress specifically, the Monsenso mobile application, which used a convergent level 4 method (proxy reported measurement tool (PSS10)) as comparison in free-living setting. The authors reported validity evidence of a linear mixed-effect regression model, which showed a statistically significant positive correlation (*P* = 0.001) between self-assessed stress and the PSS. The outcome could not be interpreted in the same way as the other studies in this review, but was added as a comparison with regards to the level of evidence for validity for tools measuring psychological stress. Table F provides a summary of self-assessment tools that measure psychological stress and their validity evidence.

**Table F:** Summary table of validity evidence of the self-assessment tools for measuring psychological stress [56,57].

#### 3.4.4 Validity of tools measuring multiple lifestyle domains

The only study that examined the validity of a measurement tool that assessed multiple lifestyle domains (PA, nutritional intake, and psychological stress)—the Ohmage platform—used a convergent level 4 method as a comparison (self-report questionnaires). The validation study was performed in free-living setting. Anthropometric bio measures and bloodspot biomarkers (collected with more than seven days in between measurements) were also described as a reference method, but have no sustainable evidence to be referred to as suitable reference methods. The validity outcomes were all weak (all correlation values < 0.60). The validity evidence of the Ohmage platform is presented in Tables D, Table E, Table F, split up over the lifestyle domains examined by the platform to enable comparison.

#### 3.4.5 Reliability

Reliability was assessed in five studies: three studies focused on PA self-assessment tools, investigating inter-device, intra-device, and test-retest reliability; one study examined the reproducibility of a nutrition self-assessment tool; one study explored the inter-method reliability of self-assessment tools covering multiple lifestyle domains. Inter-device reliability was examined for two models (HJ-151 and HJ-720ITC) of the Omron Healthcare pedometer, and inter-device reliability evidence was established (all CoV values < 2.1%); no significant differences were revealed by repeated measures ANOVA. Intra-device reliability was examined for the mobile application Pedometer FREE GPS+ regarding wear positions of the mobile device in six different environments: on the right chest and in the pants’ left pocket. Reliability was reported by the authors as ‘fair’ for the chest position (ICC = 0.72) and ‘poor’ for the pants pocket position (ICC = 0.61). Test-retest was examined for the BeWell24 mobile application which measures SB. A moderate agreement for time spent in SB, LPA, and MVPA (ICC = 0.65 [0.43, 0.82], 0.67 [0.44, 0.83], and 0.69 [0.48, 0.84], respectively) was reported by the authors. Reproducibility was examined for the Vitamin D Calculator mobile application with ICC’s and a Wilcoxon signed-rank test. It was reported that mean vitamin D and calcium intakes did not differ significantly between two measurement points (day 1 and day 3), and intraclass correlation analysis for vitamin D and calcium intakes resulted in ICC = 0.40 [0.14-0.61] and ICC = 0.22 [-0.06-0.47], respectively. The study examining the validity of the Ohmage platform compared the inter-method reliability between smartphone-based EMAs and diary reports, and their corresponding recall reports. The reliability was reported as ‘moderate’ for stress and diet (Pearson product-moment correlations ranged from 0.27 to 0.52 (*P* < .05)), and 'low' for PA (no significant associations were observed).

## 4 Discussion

This review examined the validity evidence for measurement tools used by adults to self-assess cardiovascular risk behaviors. The findings reveal that few studies have compared these tools with the corresponding conventional gold standard, and even fewer have been conducted in free-living conditions that closely replicate real-world use. Moreover, the overall results indicated suboptimal validity. There is a clear need for additional validation studies using appropriate comparison measures to provide reliable and meaningful health self-assessment data. Ultimately, medically validated self-assessment tools could enhance personalized clinical care, both in the consultation room and via telecare.

*Current state of self-assessment tools for cardiovascular risk behavior*

Most of the studies examined the validity of tools assessing a form of PA, followed by dietary assessment tools. This can be explained by the fact that, over the past decades, objective methods to measure PA have been developed, such as tracking step count and using algorithms that combine HR with accelerometery. It is noteworthy that few validation studies compare devices with a true gold standard, and even fewer are conducted in free-living conditions. The measurement of EE has proven unreliable in the vast majority of the studies, which is also demonstrated in other reviews [11,21]. Furthermore, only a limited number of devices was capable of accurately measuring step count, with most validations occurring in laboratory settings. Consequently, the validity of these devices may be even lower in free-living conditions. Clinicians in cardiac rehabilitation who wish to use such devices in practice should be aware of these findings, underlining the importance to evaluate validation studies of the devices that one intends to use clinically. To address this issue, Caiani et al. [58] have developed a standardized assessment framework that allows clinicians to verify whether sufficient validation evidence exists for the use of a mobile health solution in clinical practice. This framework can aid clinicians in selecting appropriate devices.

Self-assessment tools for HR monitoring appear to yield accurate results; however, the devices showing good validity have only been tested during constant exertion in a laboratory setting. Devices tested across various exertion levels, also in laboratory settings, showed moderate to weak validity results. Only one study investigated the validity of a HR measurement tool in free-living conditions, but only during sleep. As a result, HR measurements at different exertion levels and in free-living conditions are still unreliable. Therefore, the HR data obtained from a wrist-worn device during exercise should be interpreted with caution.

While the methods for measuring PA provide clearer metrics, measuring nutritional intake is far more complex due to the extensive variability in food composition [59]. Although direct nutritional assessment tools, such as single or multiple 24-hour recalls and (digital) food records (including those examined in this review), provide more detailed insights into dietary behaviorcompared to indirect nutritional assessment tools such as FFQs, they are less often validated due to their open-ended nature. In general, the accuracy of dietary data can be compromised at multiple stages of collection and processing, including selection bias, information bias, confounding, incorrect calculations, misclassification, and, ultimately, misleading associations [59]. Additionally, there is growing recognition of the problem of misreporting and underreporting in these methods [17]. Another reason for the limited amount of validity and reliability studies in nutritional intake assessment tools is the lack of easy-to-use and accepted gold standards for in situ assessments of these behaviors that can be readily used for objective comparison. Despite the inherent complexity and high level of effort required from the user [60], tools for monitoring nutritional intake are continually evolving, and self-reporting as a behavior change technique is increasingly supported in research. Advances in research and technology are making food composition databases more comprehensive, expanding our understanding of what constitutes ‘healthy food’, and improving the integration of technology in dietary tracking. Smartphones lighten the burden of recording nutritional intake by allowing for data entry to be readily accessible, which makes cumbersome paper diaries or PDAs obsolete. Algorithms are enhancing the accuracy of nutritional intake calculations, and some tools already use nudging techniques to encourage more immediate responses to dietary assessment prompts (such as mEMAs) to decrease recall bias. Additionally there are promising developments in the field regarding improved input methods such as meal digital photography methods, where the food intake estimate is estimated from photographs in various ways [61,62], and wearable sensors that can detect eating proxies such as chewing, swallowing, jaw movements, and hand-to-mouth gestures [60], or even a combination of these two technologies. These developments may eventually enable more accurate and objective measurement of food intake, offering the general public easy and user-friendly access to detailed information about their diet, empowering them to take informed action.

Even more challenging is measuring chronic psychological stress. Psychological stress is subjective and involves complex emotional and cognitive responses, which explains the limited evidence available for self-assessment tools in this area. One reason for this is that there is a broad array of challenges that can occur in someone’s daily life and that there are substantial individual differences in the appraisal processes, and hence the psychological stress reaction, to these stressors. This variability in exposure and responses has complicated research on stress-related factors in behavioral validation studies with regard to cardiovascular health [15]. Existing validation studies often focus on question-and-answer-based digital mental health tools used by HCPs for screening or diagnostic purposes [16], which are not suitable for self-assessment. Consequently, only two self-assessment tools for psychological stress were included in this review. Both tools employ similar methods for data collection, specifically the mEMA method. Although EMAs are not yet widely implemented in behavior analytic research, the findings of Martin-Key et al. [16] suggest that tools using mEMA methods can serve as accurate measurement tools. However, further research is needed to identify procedures and variables that enhance the accuracy of psychological stress self-assessment tools. Additionally, some studies are exploring physiological stress responses, both separately and in conjunction with subjective reports. Ongoing research in these areas could eventually lead to valid self-assessment tools for psychological stress.

Similarly, the lack of self-assessment tools for tobacco smoking and alcohol consumption can partly be explained by the complex nature of these behaviors. The tools that are currently available to measure tobacco smoking and alcohol consumption are often used in clinics or epidemiological studies [63,64] to evaluate and monitor nicotine and alcohol dependence in patients, and they involve professional guidance. They were therefore not included in the current review. Both a nicotine and alcohol dependence are influenced by a combination of biological, psychological, and social factors. Changing this behavior often requires personalized interventions, including counselling, medication, and behavioral therapy. Assessing the readiness to change these behaviors and predicting success can be complex, making it challenging to create self-assessment tools that can be used by the general public. The limited availability of validated self-assessment tools for alcohol and smoking also has implications for research and practice, as it restricts comprehensive cardiovascular risk profiling and limits the ability to evaluate or compare lifestyle interventions across all major risk behaviors.

*Validity of self-assessment tools for cardiovascular risk behavior*

In addition to the current limitations of self-assessment tools for evaluating cardiovascular risk behaviors—namely, their inability to provide a comprehensive picture of lifestyle behaviors—the validity of these measurement tools is also questionable. This review highlights that although industry-led innovations have enhanced the accuracy of consumer monitors, they still fall short compared to the best research-grade devices, and certainly do not yet measure up to the gold standards. PA measurement tools were the most extensively studied in this review, but all tools assessing EE or minutes spent in PA intensities showed weak validity. While some PA outcomes, such as step count and HR, yielded more promising results, they still do not encompass the full scope of PA, and are mostly tested in laboratory conditions instead of in free-living conditions. Similarly, the evidence for the criterion validity of tools estimating nutritional intake was moderate to weak. Many studies relied on convergent validity by comparing one measurement tool to another, which introduces significant bias into the results. Furthermore, the lack of universally accepted gold standards across domains means that criterion validity is often difficult to establish, with many comparisons relying on proxy tools rather than definitive benchmarks.

### 4.1 Strengths and weaknesses of the review

A limitation of our systematic review is the fact that search terms only focused on ‘self-evaluation’ and ‘self-assessment’. As a result, studies that examined the validity of monitoring tools, but did not mention these specific search terms, or those that involved an HCP in the monitoring/assessment process, were excluded from the search results. The latter could also be considered a strength of the review, as measurement tools such as questionnaires with complex scoring systems or interpretation should not be used by patients independently to avoid misuse.

A primary strength of this review is that we evaluated self-assessment tools for all cardiovascular risk behaviors, both risk behavior specific self-assessment tools and combined self-assessment tools. Research indicates additional benefits can be gained from tracking multiple indices of health simultaneously, to identify relationships between lifestyle domains, and thus identify potential causal mechanisms, and develop more effective interventions targeted to individual circumstances [65].

### 4.2 Implications for research

Given the importance of a healthy lifestyle in reducing cardiovascular risk, there is a clear need for more comprehensive and validated self-assessment tools. Since self-awareness and improved self-management are essential for behavior change, these tools should ideally encompass all cardiovascular risk behaviors, especially including the behaviors that are not yet covered, such as tobacco smoking [66], fostering awareness across all areas. Additionally, artificial intelligence can play a significant role in analyzing the data from these innovative tools, identifying patterns, and predicting health outcomes. Digitalization of healthcare is essential to address the growing demand for care and the declining number of HCPs. Self-assessment can enhance efficiency by allowing patients to present a comprehensive health profile to specialists, eliminating the specialists need to administer multiple assessment tools to evaluate the patients’ health. Ideally, a medical appointment could be avoided altogether if patients receive tailored and actionable advice at home, based on their health data. As such, these tools could ultimately be integrated into clinical care, making medical validation crucial. Collaboration between HCPs and patients will allow for more personalized and tailored lifestyle interventions, leading to better care, improved health outcomes, and more effective behavior change for enhanced prevention.

## 5 Conclusion

Lifestyle self-assessment tools predominantly focus on PA and nutritional intake, with limited evidence for good validity. Self-assessment tools for PA show weak validity in estimating EE and PA intensity minutes. While tools for HR and step count show better results, they are mainly evaluated in laboratory settings rather than in free-living conditions. Nutritional intake tools were mostly compared with non-criterion comparison methods, and those evaluated against criterion standards demonstrated moderate to weak validity. Furthermore, evidence on the validity of self-assessment tools for psychological stress, and for a combination of cardiovascular risk behaviors, is limited, and even absent with regards to tobacco smoking and alcohol consumption. This systematic review highlights the urgent need for the development and validation of more comprehensive and accurate self-assessment tools for cardiovascular risk behavior. Such tools hold the potential to initiate and support lifestyle behavior changes in the general population at risk for or with CVD, ultimately reducing the overall CVD burden.

## Funding

No funding was provided for this study. W.F Goevaerts was supported by the Dutch Heart Foundation, grant number 2019B015.

## PROSPERO registration number

CRD42017070945

## Availability of data and materials

All data generated or analyzed during this study are included in this published article and its supplementary information files.

## CRediT authorship contribution statement

**Wilhelmina F. Goevaerts:** Writing – original draft, Methodology, Investigation, Formal analysis, Data curation, Conceptualization. **Joyce M. Heutinck:** Writing – review & editing, Investigation, Data curation. **Mayke M.C.J. Van Leunen:** Writing – review & editing. **Wessel W. Nieuwenhuys:** Investigation, Data curation. **Lonneke A. Fruytier:** Investigation, Data curation. **Cyrille Herkert:** Investigation, Data curation. **Jos J. Kraal:** Investigation, Data curation. **Ilse A.G. Rongen:** Methodology, Data curation, Conceptualization. **Willem J. Kop:** Writing – review & editing, Supervision. **Yuan Lu:** Writing – review & editing, Supervision. **Hareld M.C. Kemps:** Writing – review & editing, Supervision. **Rutger W.M. Brouwers:** Writing – review & editing, Methodology, Data curation, Conceptualization.

## Declaration of competing interest

W.F. Goevaerts reports a relationship with Netherlands Heart Foundation that includes: funding grants. If there are other authors, they declare that they have no known competing financial interests or personal relationships that could have appeared to influence the work reported in this paper.

## Appendices

Appendix A – PRISMA 2020 main and abstract checklists

Appendix B – Search strategy and outcomes

Appendix C – Excluded studies with reasons

Appendix D – Source of funding for each study

Appendix E – QUADAS-2 risk of bias assessment for each study

## Appendix B Supplementary materials

Image, application 1

Image, application 2

Image, application 3

Image, application 4

Image, application 5
