# Supplementary material for: Validity of self-assessment tools for cardiovascular risk behaviors: A systematic review
Source: Am J Prev Cardiol. 2025 Oct 7;24:101316. doi: 10.1016/j.ajpc.2025.101316 (PMC12663659; doi:10.1016/j.ajpc.2025.101316)
Supplement: Supplementary file 2 [file mmc2.pdf]

Appendix B – Search strategy and outcomes

Ovid Embase

Exp…/ = thesaurus term including narrower terms. .ti,ab, = search in title and abstract. *= truncation

	OVID embase (1974 to 2023 week 32) **searched on August 16th 2023**	
1	exp self evaluation/ OR (self assessment* or self check* or self monitoring or self management).ab,ti.	93006
2	exp exercise/ OR exp sport/ OR exp food/ OR exp diet/ OR exp nutritional assessment/ OR exp drinking behavior/ OR exp smoking/ OR exp mental stress/ OR exp relaxation training/ OR (exercise* or physical activit* or physical inactivit* or leisure activit* or leisure time activit* or sport* OR nutrition* or food or fruit* or vegetable* or salt* or saturated fat* or unsaturated fat* OR alcohol* OR smoker* or smoking OR stress* OR relaxation*).ab,ti.	5446419
3	exp validation study/ OR (validation or validity).ab,ti.	672796
4	1 and 2 and 3	1046
5	Limit 4 to conference abstract status	244
6	4 not 5	802

PubMed

[mesh] = thesaurus term including narrower terms. [tiab] = search in title, abstracts en author keywords. *=truncation. "…"= phrase

	PubMed **searched on August 16th 2023**	
#1	("Self-Assessment"[Mesh] OR "self assessment*"[tiab] OR "self check*"[tiab] OR "self monitoring"[tiab] OR "self management"[tiab])	61223
#2	("Exercise"[Mesh] OR "Leisure Activities"[Mesh] OR "Food"[Mesh] OR "Diet"[Mesh] OR "Nutrition Assessment"[Mesh] OR "Alcohol Drinking"[Mesh] OR "Smoking"[Mesh] OR "Stress, Psychological"[Mesh] OR "Relaxation Therapy"[Mesh] OR exercise*[tiab] OR "physical activit*"[tiab] OR "physical inactivit*"[tiab] OR "Leisure Activit*"[tiab] OR "leisure time activit*"[tiab] OR sport*[tiab] OR nutrition*[tiab] OR food*[tiab] OR fruit*[tiab] OR vegetable*[tiab] OR salt*[tiab] OR "saturated fat*"[tiab] OR "unsaturated fat*"[tiab] OR alcohol*[tiab] OR smoker*[tiab] OR smoking*[tiab] OR stress*[tiab] OR relaxation*[tiab])	4249280
#3	("Validation Studies as Topic"[Mesh] OR "Validation Study"[Publication Type] OR validation[tiab] OR validity[tiab])	530805
#4	#1 AND #2 AND #3	797

Cochrane

MeSH descriptor = thesaurus term including narrower terms. :ti,ab,kw = search in title, abstracts en author keywords. *=truncation.

	Cochrane library **searched on August 16th 2023**	

#1	MeSH descriptor: [Self-Assessment] explode all trees	833
#2	((self assessment) OR (self check) OR (self monitoring) OR (self management)):ti,ab,kw (Word variations have been searched)	89012
#3	#1 or #2	89012
#4	MeSH descriptor: [Exercise] explode all trees	38402
#5	MeSH descriptor: [Leisure Activities] explode all trees	25102
#6	MeSH descriptor: [Food] explode all trees	45334
#7	MeSH descriptor: [Diet] explode all trees	25742
#8	MeSH descriptor: [Nutrition Assessment] explode all trees	856
#9	MeSH descriptor: [Alcohol Drinking] explode all trees	5129
#10	MeSH descriptor: [Smoke] explode all trees	634
#11	MeSH descriptor: [Stress, Psychological] explode all trees	6355
#12	MeSH descriptor: [Relaxation Therapy] explode all trees	2549
#13	((exercise) OR (sport) OR (physical activity) OR (physical inactivity) OR (leisure activity) or (nutrition) OR (food) OR (diet) OR (fruit) OR (vegetable) OR (salt) OR (saturated fat) OR (unsaturated fat) OR (alcohol) OR (smoking) OR (smoker) OR (stress) OR (relaxation)):ti,ab,kw	384327
#14	#4 or #5 or #6 or #7 or #8 or #9 or #10 or #11 or #12 or #13	408087
#15	MeSH descriptor: [Validation Study] explode all trees	161
#16	MeSH descriptor: [Validation Studies as Topic] explode all trees	44
#17	((validation) OR (validity)):ti,ab,kw	27679
#18	#15 or #16 or #17	27679
#19	#3 and #14 and #18	1322
