# Supplementary material for: Validity of self-assessment tools for cardiovascular risk behaviors: A systematic review
Source: Am J Prev Cardiol. 2025 Oct 7;24:101316. doi: 10.1016/j.ajpc.2025.101316 (PMC12663659; doi:10.1016/j.ajpc.2025.101316)
Supplement: Supplementary file 3 [file mmc3.pdf]

Appendix C – Excluded studies with reasons

Total excluded following full paper screening (*n* = 127)

Exclusion criteria B – Publication not in English (*n* = 1)

1. Huang K, Li M, Shi L, Zheng Z, Wen F, Zhang Y. Validity and reliability of Chinese version of Occupational Sitting and Physical Activity Questionnaire among intensive care nurses. *Journal of Shanghai Jiaotong University (Medical Science)* 2022;**42**:1582–1588.

Exclusion criteria C - Comment, editorial, letter, news, congress, or protocol (*n* = 4)

1. Boateng G, Batsis JA, Halter R, Kotz D. ActivityAware: An App for Real-Time Daily Activity Level Monitoring on the Amulet Wrist-Worn Device. *Proceedings of the .IEEE International Conference on Pervasive Computing and Communications Workshops : PerCom .IEEE International Conference on Pervasive Computing and CommunicationsWorkshops* 2017;**2017**:10.1109/PERCOMW.2017.7917601. Epub 2017 May 4-10.1109/PERCOMW.2017.7917601. Epub 2017 May 4.
2. Dutton S, Dennis S, Harris M, Zwar N, Bauman A, Der PHV. Feasibility and acceptability of two instruments for measuring physical activity (PA) in primary care. 2012;**15**.
3. Nordgren B, Friden C, Jansson E, Osterlund T, Grooten WJA, Opava CH, Rickenlund A. Validation of the fox-walk test in people with rheumatoid arthritis. 2013;**65**.
4. Sousa SRD, Bourbeau J, Li PZ, Ahmed S. Validtiy and usability testing of the Fitbit pedometer in patients with COPD. 2015;**46**.

Exclusion criteria F - Children or adolescents (*n* = 1)

1. Wyse J, Mercer T, Ashford B, Buxton K, Gleeson N. Evidence for the validity and utility of the stages of exercise behaviour change scale in young adults. 1995:365–365.

Exclusion criteria H - Intervention: No cardiovascular risk behavior (i.e., physical activity, tobacco smoking, alcohol consumption, nutritional intake, or psychological stress) (*n* = 23)

1. Bai J. Development and validation of the Acculturative Stress Scale for Chinese College Students in the United States (ASSCS). *Psychological assessment* 2016;**28**:443–443.
2. Beebe R, Frisch N. Development of the Differentiation of Self and Role Inventory for Nurses (DSRI-RN): a tool to measure internal dimensions of workplace stress. *Nursing outlook* 2009;**57**:240–240.
3. Book K, Marten-Mittag B, Henrich G, Dinkel A, Scheddel P, Sehlen S, Haimerl W, Schulte T, Britzelmeir I, Herschbach P. Distress screening in oncology - Evaluation of

the Questionnaire on Distress in Cancer Patients - Short form (QSC-R10) in a German sample. 2011:287–287.
4. Brown LJ, Adlam T, Hwang F, Khadra H, Maclean LM, Rudd B, Smith T, Timon C, Williams EA, Astell AJ. Computerized Self-Administered Measures of Mood and Appetite for Older Adults. *Journal of applied gerontology : the official journal of the Southern Gerontological Society* 2016:733464816630636–733464816630636.
5. Cawood AL, Elia M, Sharp SKE, Stratton RJ. Malnutrition self-screening by using MUST in hospital outpatients: Validity, reliability, and ease of use. 2012:1000–1000.
6. Christo G, Jones SL, Haylett S, Stephenson GM, Lefever RM, Lefever R. The Shorter PROMIS Questionnaire: further validation of a tool for simultaneous assessment of multiple addictive behaviours. *Addict Behav* 2003;**28**:225–248.
7. Currie SR. Confirmatory factor analysis of the reasons for smoking scale in alcoholics. 2004:465–465.
8. Dyrbye LN, Schwartz A, Downing SM, Szydlo DW, Sloan JA, Shanafelt TD. Efficacy of a brief screening tool to identify medical students in distress. *Academic medicine : journal of the Association of American Medical Colleges* 2011;**86**:907–914.
9. Fiala KA, D'Abundo ML, Marinaro LM. Construct validity and reliability of college students' responses to the reasons for smoking scale. *Journal of American college health : J of ACH* 2010;**58**:571–577.
10. Fitzsimmons-Craft EE, Bardone-Cone AM, Harney MB. Development and validation of the Body, Eating, and Exercise Comparison Orientation Measure (BEECOM) among college women. *Body image* 2012;**9**:476–476.
11. Frank SH, Graham AV, Zyzanski SJ, White S. Use of the Family CAGE in screening for alcohol problems in primary care. 1992:209–209.
12. Graue M, Haugstvedt A, Wentzel-Larsen T, Iversen MM, Karlsen B, Rokne B. Diabetes-related emotional distress in adults: reliability and validity of the Norwegian versions of the Problem Areas in Diabetes Scale (PAID) and the Diabetes Distress Scale (DDS). *International journal of nursing studies* 2012;**49**:174–182.
13. Joensen LE, Tapager I, Willaing I. Validation of the Danish version of diabetes distress scale. 2012;**55**.
14. Klitzke M, Irwin R, Lombardo TW, Christoff KA. Self-monitored smoking motives. 1990:121–121.
15. Mo C, Deane FP, Lyons GC, Kelly PJ. Factor analysis and validity of a short six-item version of the Desires for Alcohol Questionnaire. *Journal of substance abuse treatment* 2013;**44**:557–564.
16. Morasso G, Costantini M, Baracco G, Borreani C, Capelli M. Assessing psychological distress in cancer patients: validation of a self-administered questionnaire. *Oncology* 1996;**53**:295–302.
17. Nakawatase Y, Taru C, Tsutou A, Shiotani H, Kido Y, Ohara T, Ogawa W, Miyawaki I. Development of an evaluation scale for self-management behavior related to physical activity of type 2 diabetic patients. 2007:2843–2843.
18. Nordgren B, Friden C, Jansson E, Osterlund T, Grooten WJ, Opava CH, Rickenlund A. Criterion validation of two submaximal aerobic fitness tests, the self-monitoring Fox-walk test and the Astrand cycle test in people with rheumatoid arthritis. *BMC musculoskeletal disorders* 2014;**15**:305–305.
19. O'Farrell TJ, Fals-Stewart W, Murphy M. Concurrent validity of a brief self-report Drug Use Frequency measure. *Addict Behav* 2003;**28**:327–337.

20. Otani T, Yoshii C, Kano M, Kitada M, Inagaki K, Kurioka N, Isomura T, Hara M, Okubo Y, Koyama H. Validity and Reliability of Kano Test for Social Nicotine Dependence. 2009:815–815.
21. Pinto AM, Fava JL, Raynor HA, LaRose JG, Wing RR. Development and validation of the weight control strategies scale. *Obesity (Silver Spring, Md)* 2013;**21**:2429–2436.
22. Tokunaga-Nakawatase Y, Taru C, Miyawaki I. Development of an evaluation scale for self-management behavior related to physical activity of patients with coronary heart disease. *European journal of cardiovascular nursing : journal of the Working Group on Cardiovascular Nursing of the European Society of Cardiology* 2012;**11**:168–168.
23. Tso IF, Grove TB, Taylor SF. Self-assessment of psychological stress in schizophrenia: Preliminary evidence of reliability and validity. *Psychiatry research* 2012;**195**:39–44.

Exclusion criteria I - Intervention: No self-assessment: cannot be filled in by patient/person individually (without support or help from a professional) (*n* = 73)

1. Ahmad S, Harris T, Limb E, Kerry S, Victor C, Ekelund U, Iliffe S, Whincup P, Beighton C, Ussher M, Cook DG. Evaluation of reliability and validity of the General Practice Physical Activity Questionnaire (GPPAQ) in 60-74 year old primary care patients. *BMC family practice* 2015;**16**:8–8.
2. Akohoue SA, Wallston KA, Schlundt DG, Rothman RL. Psychometric evaluation of the short version of the Personal Diabetes Questionnaire to assess dietary behaviours and exercise in patients with type 2 diabetes. 2017;**26**:182–182.
3. Alvarez-Gallardo IC, Soriano-Maldonado A, Segura-Jimenez V, Carbonell-Baeza A, Estevez-Lopez F, McVeigh JG, Delgado-Fernandez M, Ortega FB. International FItness Scale (IFIS): Construct Validity and Reliability in Women With Fibromyalgia: The al-Andalus Project. *Archives of Physical Medicine and Rehabilitation* 2016;**97**:395–404.
4. Amirkhan JH, Urizar GG, Clark S. Criterion validation of a stress measure: The Stress Overload Scale. 2015:985–985.
5. Backhouse MR, Hensor EM, White D, Keenan AM, Helliwell PS, Redmond AC. Concurrent validation of activity monitors in patients with rheumatoid arthritis. *Clinical biomechanics (Bristol, Avon)* 2013;**28**:473–479.
6. Baer HJ, Blum RE, Rockett HR, Leppert J, Gardner JD, Suitor CW, Colditz GA. Use of a food frequency questionnaire in American Indian and Caucasian pregnant women: a validation study. *BMC public health* 2005;**5**:135–135.
7. Beliard S, Coudert M, Valero R, Charbonnier L, Duchene E, Allaert FA, Bruckert E. Validation of a short food frequency questionnaire to evaluate nutritional lifestyles in hypercholesterolemic patients. *Annales d'Endocrinologie* 2012;**73**:523–529.
8. Berthouze SE, Minaire PM, Chatard J-C, Boutet C, Castells J, Lacour -. J R. A new tool for evaluating energy expenditure: The 'QAPSE' development and validation. 1993:1405–1405.
9. Block G, Thompson FE, Hartman AM, Larkin FA, Guire KE. Comparison of two dietary questionnaires validated against multiple dietary records collected during a 1-year period. 1992:686–686.

10. Bond JC, Greenfield TK, Patterson D, Kerr WC. Adjustments for drink size and ethanol content: new results from a self-report diary and transdermal sensor validation study. *Alcoholism, Clinical and Experimental Research* 2014;**38**:3060–3067.
11. Calabro MA, Welk GJ, Carriquiry AL, Nusser SM, Beyler NK, Mathews CE, Matthews CE. Validation of a computerized 24-hour physical activity recall (24PAR) instrument with pattern-recognition activity monitors. *Journal of physical activity & health* 2009;**6**:211–220.
12. Camoes M, Severo M, Santos AC, Barros H, Lopes C. Testing an adaptation of the EPIC physical activity questionnaire in Portuguese adults: a validation study that assesses the seasonal bias of self-report. *Annals of Human Biology* 2010;**37**:185–185.
13. Chasan-Taber L, Schmidt MD, Roberts DE, Hosmer D, Markenson G, Freedson PS. Development and validation of a pregnancy physical activity questionnaire. 2004:1750–1750.
14. Darviri C, Alexopoulos EC, Artemiadis AK, Tigani X, Kraniotou C, Darvyri P, Chrousos GP. The Healthy Lifestyle and Personal Control Questionnaire (HLPCQ): a novel tool for assessing self-empowerment through a constellation of daily activities. *BMC public health* 2014;**14**:995–995.
15. Devereaux MK, Williamson E, Futrell M, Chamberlain C. A self-assessment tool to measure older adults' perceptions regarding physical fitness and exercise activity. 1997:1220–1220.
16. Dobson AJ, Blijlevens R, Alexander HM, Croce N, Heller RF, Higginbotham N, Pike G, Plotnikoff R, Russell A, Walker R. Short fat questionnaire: A self-administered measure of fat-intake behaviour. 1993:144–144.
17. Feuerlein W, Ringer C, Kufner H, Antons K. Diagnosis of alcoholism: the Munich Alcoholism Test (MALT). 1979;**7**:137–137.
18. Fidanza F, Gentile MG, Porrini M. A self-administered semiquantitative food-frequency questionnaire with optical reading and its concurrent validation. *Eur J Epidemiol* 1995;**11**:163–170.
19. Flegal KM, Larkin FA, Metzner HL, Thompson FE, Guire KE. Counting calories: Partitioning energy intake estimates from a food frequency questionnaire. 1988:749–749.
20. Francis H, Stevenson R. Validity and test-retest reliability of a short dietary questionnaire to assess intake of saturated fat and free sugars: a preliminary study. *Journal of human nutrition and dietetics : the official journal of the British Dietetic Association* 2013;**26**:234–234.
21. Gilsing A, Mayhew AJ, Payette H, Shatenstein B, Kirkpatrick SI, Amog K, Wolfson C, Kirkland S, Griffith LE, Raina P. Validity and reliability of a short diet questionnaire to estimate dietary intake in older adults in a subsample of the Canadian longitudinal study on aging. *Nutrients* 2018;**10**:1522.
22. Giovannucci E, Colditz G, Stampfer MJ, Rimm EB, Litin L, Sampson L, Willett WC. The assessment of alcohol consumption by a simple self-administered questionnaire. 1991:810–810.
23. Gosney JL, Scott JA, Snook EM, Motl RW. Physical activity and multiple sclerosis: validity of self-report and objective measures. *Family & community health* 2007;**30**:144–150.

24. Groenland EH, Vendeville JAC, Bots ML, Visseren FLJ, Musson REA, Spiering W. Validation of spot urine in estimating 24-h urinary sodium, potassium and sodium-to-potassium ratio during three different sodium diets in healthy adults. *Blood Press* 2023;**32**:2170868.
25. Heesch KC, Hill RL, Uffelen JG van, Brown WJ. Are Active Australia physical activity questions valid for older adults? *Journal of science and medicine in sport* 2011;**14**:233–237.
26. Jin S, Cong M, Zhang L, Wang Y, Qin D, Lu Q. Validation of a simple diet self-assessment tool (SDSAT) in head and neck cancer patients undergoing radiotherapy. *Eur J Oncol Nurs* 2020;**44**:101702.
27. Johnson MF, Sallis JF, Hovell MF. Self-report assessment of walking: Effects of aided recall instructions and item order. 2000:141–141.
28. Johnston EA, Park A, Hu L, Yi SS, Thorpe LE, Rummo PE, Beasley JM. Relative validity of a Diet Risk Score (DRS) for Chinese American adults. *BMJ Nutrition, Prevention and Health* 2023;**6**:76–82.
29. Kalisch T, Theil C, Gosheger G, Schwarze J, Voss K, Schoenhals I, Moellenbeck B. Validation of a Modified Version of the German Sedentary Behavior Questionnaire. *Healthcare (Basel)* 2022;**10**.
30. Kato S, Waki K, Nakamura S, Osada S, Kobayashi H, Kadowaki T, Ohe K, Fujita H. Validation of using photos to evaluate dietary intake-the method used by dialbetics, a smartphone-based selfmanagement system for diabetes patients. 2015;**17**.
31. Klesges RC, Klesges LM, Swenson AM, Pheley AM. A validation of two motion sensors in the prediction of child and adult physical activity levels. *American Journal of Epidemiology* 1985;**122**:400–410.
32. Knapik JJ, Jones BH, Reynolds KL, Staab JS. Validity of self-assessed physical fitness. 1992:367–367.
33. Król-Zielińska M, Ciekot-Sołtysiak M, Szeklicki R, Zieliński J, Osiński W, Kantanista A. Validity and Reliability of the Polish Adaptation of the CHAMPS Physical Activity Questionnaire. *Biomed Res Int* 2019;**2019**:6187616.
34. Kupetz K, Klagsbrun M, Wisoff D. The acceptance and validity of the substance use and abuse survey (SUAS). 1979:163–163.
35. Leving MT, Horemans HLD, Vegter RJK, Groot S de, Bussmann JBJ, Woude LHV van der. Validity of consumer-grade activity monitor to identify manual wheelchair propulsion in standardized activities of daily living. *PLoS One* 2018;**13**:e0194864.
36. Lim LS, Williams DE, Hagen PT. Validation of a five-point self-rated stress score. *Am J Health Promot* 2005;**19**:438–441.
37. Lindroos A-K, Lissner L, Sjostrom L. Validity and reproducibility of a self-administered dietary questionnaire in obese and non-obese subjects. 1993:461–461.
38. Little P, Barnett J, Margetts B, Kinmonth A-LL, Gabbay J, Thompson R, Warm D, Warwick H, Wooton S. The validity of dietary assessment in general practice. *Journal of epidemiology and community health* 1999;**53**:165–165.
39. Liu L, Wang PP, Roebothan B, Ryan A, Tucker CS, Colbourne J, Baker N, Cotterchio M, Yi Y, Sun G. Assessing the validity of a self-administered food-frequency questionnaire (FFQ) in the adult population of Newfoundland and Labrador, Canada. 2013.
40. Lönn A, Kallings LV, Börjesson M, Ekblom Ö, Ekström M. Convergent validity of commonly used questions assessing physical activity and sedentary time in Swedish patients after myocardial infarction. *BMC Sports Sci Med Rehabil* 2022;**14**:117.

41. Maes I, Ketels M, Van Dyck D, Clays E. The occupational sitting and physical activity questionnaire (OSPAQ): a validation study with accelerometer-assessed measures. *BMC Public Health* 2020;**20**:1072.
42. McCullough J, Keller H. The My Meal Intake Tool (M-MIT): Validity of a patient self-assessment for food and fluid intake at a single meal. 2016:1–1.
43. McNaughton SA, Marks GC, Gaffney P, Williams G, Green A. Validation of a food-frequency questionnaire assessment of carotenoid and vitamin E intake using weighed food records and plasma biomarkers: The method of triads model. 2005:211–211.
44. Midanik L. Over-reports of recent alcohol consumption in a clinical population: A validity study. 1982:101–101.
45. Monni A, Scalas LF. Health Risk Behaviour Inventory Validation and its Association with Self-regulatory Dispositions. *Journal of Clinical Psychology in Medical Settings* 2022;**29**:861–874.
46. Murakami K, Sasaki S, Takahashi Y, Okubo H, Hirota N, Notsu A, Fukui M, Date C. Reproducibility and relative validity of dietary glycaemic index and load assessed with a self-administered diet-history questionnaire in Japanese adults. 2008:639–639.
47. Napolitano MA, Borradaile KE, Lewis BA, Whiteley JA, Longval JL, Parisi AF, Albrecht AE, Sciamanna CN, Jakicic JM, Papandonatos GD, Marcus BH. Accelerometer use in a physical activity intervention trial. *Contemporary clinical trials* 2010;**31**:514–523.
48. Parker DR, Lasater TM, Windsor R, Wilkins J, Upegui DI, Heimdal J. The accuracy of self-reported smoking status assessed by cotinine test strips. *Nicotine Tob Res* 2002;**4**:305–309.
49. Pickett KE, Rathouz PJ, Kasza K, Wakschlag LS, Wright R. Self-reported smoking, cotinine levels, and patterns of smoking in pregnancy. *Paediatr Perinat Epidemiol* 2005;**19**:368–376.
50. Purushothama J, Badiger S, Kumar N, Baikunje N, D'Souza N, D'Mello M, Olickal JJ. Validity assessment of self-reported smoking status to detection of urine cotinine levels among patients with tuberculosis undergoing tobacco cessation treatment. *Biomedicine (India)* 2022;**42**:371–376.
51. Roberts-Lewis SF, White CM, Ashworth M, Rose MR. The validity of the International Physical Activity Questionnaire (IPAQ) for adults with progressive muscle diseases. *Disabil Rehabil* 2022;**44**:7312–7320.
52. Rohrmann S, Klein G. Validation of a short questionnaire to qualitatively assess the intake of total fat, saturated, monounsaturated, polyunsaturated fatty acids, and cholesterol. 2003:111–111.
53. Sakai JT, Mikulich-Gilbertson SK, Long RJ, Crowley TJ. Validity of transdermal alcohol monitoring: fixed and self-regulated dosing. *Alcoholism, Clinical and Experimental Research* 2006;**30**:26–33.
54. Sakata S, Tsuchihashi T, Oniki H, Tominaga M, Arakawa K, Sakaki M, Kitazono T. Relationship between salt intake as estimated by a brief self-administered diet-history questionnaire (BDHQ) and 24-h urinary salt excretion in hypertensive patients. 2015:560–560.
55. Sanchez-Craig M, Annis HM. 'Self-monitoring' and 'recall' measures of alcohol consumption: Convergent validity with biochemical indices of liver function. 1982:117–117.

56. Sasaki S, Ushio F, Amano K, Morihara M, Todoriki T, Uehara Y, Toyooka T. Serum biomarker-based validation of a self-administered diet history questionnaire for Japanese subjects. 2000:285–285.
57. Shaffer HJ, Eber GB, Hall MN, Vander Bilt J. Smoking behavior among casino employees: self-report validation using plasma cotinine. *Addict Behav* 2000;**25**:693–704.
58. Shatenstein B, Xu H, Luo ZC, Fraser W. Relative validity of a food frequency questionnaire for pregnant women. *Revue canadienne de la pratique et de la recherche en dietetique [Canadian journal of dietetic practice and research]* 2011;**72**:60–69.
59. Siebeling L, Wiebers S, Beem L, Puhan MA, ter RG. Validity and reproducibility of a physical activity questionnaire for older adults: Questionnaire versus accelerometer for assessing physical activity in older adults. 2012:171–171.
60. Simons JS, Wills TA, Emery NN, Marks RM. Quantifying alcohol consumption: Self-report, transdermal assessment, and prediction of dependence symptoms. *Addictive Behaviors* 2015;**50**:205–205.
61. Singh A, Babyak MA, Brummett BH, Jiang R, Watkins LL, Barefoot JC, Kraus WE, Shah SH, Siegler IC, Hauser ER, Williams RB. Computing a Synthetic Chronic Psychosocial Stress Measurement in Multiple Datasets and its Application in the Replication of G x E Interactions of the EBF1 Gene. 2015:489–489.
62. Snook EM, Motl RW, Gliottoni RC. The effect of walking mobility on the measurement of physical activity using accelerometry in multiple sclerosis. *Clinical rehabilitation* 2009;**23**:248–258.
63. Spencer EH, Elon LK, Hertzberg VS, Stein AD, Frank E. Validation of a brief diet survey instrument among medical students. 2005:802–802.
64. Stroyer J, Essendrop M, Jensen LD, Warming S, Avlund K, Schibye B. Validity and reliability of self-assessed physical fitness using visual analogue scales. *Perceptual and motor skills* 2007;**104**:519–533.
65. Timperio A, Salmon J, Crawford D. Validity and reliability of a physical activity recall instrument among overweight and non-overweight men and women. 2003:477–477.
66. Uchalik DC. A comparison of questionnaire and self-monitored reports of alcohol intake in a nonalcoholic population. 1979:409–409.
67. Vanroy C, Vanlandewijck Y, Cras P, Feys H, Truijen S, Michielsen M, Vissers D. Is a coded physical activity diary valid for assessing physical activity level and energy expenditure in stroke patients? *PloS one* 2014;**9**:e98735–e98735.
68. Washburn RA, Smith KW, Jette AM, Janney CA. The Physical Activity Scale for the Elderly (PASE): development and evaluation. *Journal of clinical epidemiology* 1993;**46**:153–162.
69. Washburn RA, Zhu W, McAuley E, Frogley M, Figoni SF. The physical activity scale for individuals with physical disabilities: Development and evaluation. 2002:193–193.
70. Weening-Dijksterhuis E, de GM, Krijnen W, der SC van, Greef MH de, Krijnen W, Schans CP van der. Self-reported physical fitness in frail older persons: Reliability and validity of the self-assessment of physical fitness (SAPF). *Perceptual and motor skills* 2012;**115**:797–797.
71. Weiss TW, Slater CH, Green LW, Kennedy VC, Albright DL, Wun C-C. The validity of single-item, self-assessment questions as measures of adult physical activity. 1990:1123–1123.

72. Williams JG, Purewal RS. Development and initial validation of the effort sense rating scale (ESRS): A self-perceived index of physical fitness. 2001:103–103.
73. Wilson JS, Elborn JS, Fitzsimons D, McCrum-Gardner E. Do smokers with chronic obstructive pulmonary disease report their smoking status reliably? A comparison of self-report and bio-chemical validation. *International journal of nursing studies* 2011;**48**:856–862.

Exclusion criteria K - No validation study (*n* = 14)

1. Anton RF, Moak DH, Latham P. The obsessive compulsive drinking scale: A self-rated instrument for the quantification of thoughts about alcohol and drinking behavior. 1995:92–92.
2. Arakawa M, Watanabe T, Suzuki K, Nishino J, Sekizuka H, Iwahori T, Ono SI, Hidaka S. Validation of Self-Monitoring Devices Supporting Sodium Intake Reduction: An Experimental Feeding Study Using Standardized Low-Salt and High-Salt Meals among Healthy Japanese Volunteers. *Ann Nutr Metab* 2021;**77**:289–298.
3. Arheart KL, Lee DJ, Fleming LE, LeBlanc WG, Dietz NA, McCollister KE, Wilkinson JD, Lewis JE, 3rd JDC, Davila EP, Bandiera FC, Erard MJ. Accuracy of self-reported smoking and secondhand smoke exposure in the US workforce: the National Health and Nutrition Examination Surveys. *Journal of occupational and environmental medicine* 2008;**50**:1414–1420.
4. Denollet J. Emotional distress and fatigue in coronary heart disease: The global mood scale (GMS). *Psychological Medicine* 1993;**23**:111–121.
5. Forney KJ, Holland LA, Joiner TE, Keel PK. Determining empirical thresholds for ‘definitely large’ amounts of food for defining binge-eating episodes. *Eating disorders* 2015;**23**:15–30.
6. Masood K, Ahmed B, Choi J, Gutierrez-Osuna R. Consistency and validity of self-reporting scores in stress measurement surveys. 2012;**2012**:4895–4895.
7. McNaughton SA, Ball K, Crawford D, Mishra GD. An index of diet and eating patterns is a valid measure of diet quality in an Australian population. 2008:86–86.
8. Morean ME, Corbin WR. Subjective alcohol effects and drinking behavior: the relative influence of early response and acquired tolerance. *Addictive Behaviors* 2008;**33**:1306–1313.
9. Muaremi A, Arnrich B, Troster G. Towards Measuring Stress with Smartphones and Wearable Devices During Workday and Sleep. 2013:172–172.
10. Ohkawara K, Oshima Y, Hikihara Y, Ishikawa-Takata K, Tabata I, Tanaka S. Real-time estimation of daily physical activity intensity by a triaxial accelerometer and a gravity-removal classification algorithm. *The British journal of nutrition* 2011;**105**:1681–1681.
11. Perez-Padilla J, Menendez S, Lozano O. Validity of the Parenting Stress Index Short Form in a Sample of At-Risk Mothers. *Evaluation review* 2015;**39**:428–446.
12. Pirie PL, Luepker RV, Jr. DRJ. Development and validation of a self-scoring test for coronary heart disease risk. 1983:65–65.
13. Salley JN, Hoover AW, Wilson ML, Muth ER. Comparison between Human and Bite-Based Methods of Estimating Caloric Intake. *Journal of the Academy of Nutrition and Dietetics* 2016;**116**:1568–1577.
14. Stubbs RJ, O’Reilly LM, Whybrow S, Fuller Z, Johnstone AM, Livingstone MBE, Ritz P, Horgan GW. Measuring the difference between actual and reported food intakes

in the context of energy balance under laboratory conditions. *British journal of nutrition* 2014;**111**:2032–2043.

Exclusion criteria L - No index- and/or reference-test described (*n* = 11)

1. Ann YB, Johnson RK, Harvey-Berino J, Gold BC, Yon BA, Johnson RK, Harvey-Berino J, Gold BC. The Use of a Personal Digital Assistant for Dietary Self-Monitoring Does Not Improve the Validity of Self-Reports of Energy Intake. *Journal of the American Dietetic Association* 2006;**106**:1256–1259.
2. Cooney NL, Meyer RE, Kaplan RF, Baker LH. A validation study of four scales measuring severity of alcohol dependence. *Br J Addict* 1986;**81**:223–229.
3. Davis Jr LJ, Hurt RD, Offord KP, Lauger GG, Morse RM, Bruce BK. Self-administered Nicotine-Dependence Scale (SANDS): item selection, reliability estimation, and initial validation. *J Clin Psychol* 1994;**50**:918–930.
4. Donini LM, Marrocco W, Marocco C, Lenzi A. Validity of the Self-Mini Nutritional Assessment (Self-MNA) for the Evaluation of Nutritional Risk. A Cross-Sectional Study Conducted in General Practice. *Journal of Nutrition, Health and Aging* 2018;**22**:44–52.
5. Fuller RK, Lee KK, Gordis E. Validity of self-report in alcoholism research: results of a Veterans Administration Cooperative Study. *Alcohol Clin Exp Res* 1988;**12**:201–205.
6. Hezarjaribi N, Mazrouee S, Ghasemzadeh H. Speech2Health: A Mobile Framework for Monitoring Dietary Composition From Spoken Data. *IEEE J Biomed Health Inform* 2018;**22**:252–264.
7. Joffe R, Lowe MR, Jr. EBF. A validity test of the reasons for smoking scale. 1981:41–41.
8. Mitchell DT, Korslund MK, Brewer BK, Novascone MA. Development and validation of the Cholesterol-Saturated Fat Index (CSI) Scorecard: A dietary self-monitoring tool. 1996:132–132.9.
9. Motl RW, McAuley E, Snook EM, Scott JA. Validity of physical activity measures in ambulatory individuals with multiple sclerosis. 2006:1151–1151.
10. Newman-Beinart NA, Norton S, Dowling D, Gavriloff D, Vari C, Weinman JA, Godfrey EL. The development and initial psychometric evaluation of a measure assessing adherence to prescribed exercise: The Exercise Adherence Rating Scale (EARS). 2017:180–180.
11. Ozasa K, Higashi A, Yamasaki M, Hayashi K, Watanabe Y. Validity of observed differences in dietary surveys by two self- administered questionnaires over a 5-year period: Concordance with self- reported change. 1997:85–85.
