# Supplementary material for: Validity of self-assessment tools for cardiovascular risk behaviors: A systematic review
Source: Am J Prev Cardiol. 2025 Oct 7;24:101316. doi: 10.1016/j.ajpc.2025.101316 (PMC12663659; doi:10.1016/j.ajpc.2025.101316)
Supplement: Supplementary file 4 [file mmc4.pdf]

Appendix D – Source of funding for each study

Study details	Source of funding
**Studies evaluating self-assessment tools assessing physical activity (*n* = 22)**	
Åkerberg et al., 2016, Sweden, [30]	This work was supported by the Knowledge Foundation (KKS)(grant number 20120275).
Chowdhury et al., 2017, UK, [5]	This study was funded in part through grant (MR/J00040X/1) from the National Prevention Research Initiative (NPRI) to DT. Funding partners are: Alzheimer's Research Trust, Alzheimer's Society, Biotechnology and Biological Sciences Research Council, British Heart Foundation; Cancer Research UK; Chief Scientists Office, Scottish Government Health Directorate; Department of Health; Diabetes UK; Economic and Social Research Council; Health and Social Care Research and Development Division of the Public Health Agency; Medical Research Council; The Stroke Association; Wellcome Trust; Welsh Assembly Government and World Cancer Research Fund. The funders had no role in study design, data collection and analysis, decision to publish, or preparation of the manuscript.
Dooley et al., 2017, USA, [31]	No funding was provided for this study.
Gomersall et al., 2016, Australia, [32]	Drs Gomersall and Pavey were supported by an Australian National Health and Medical Research Council (NHMRC) program grant (NHMRC no: 569940). The study was funded by a Start-Up Grant from The University of Queensland.
Holbrook et al., 2009, USA, [33]	None declared.
Leth et al., 2017, Denmark, [34]	None declared.
Middelweerd et al., 2017, The Netherlands, [35]	This research is supported by Philips and Technology Foundation STW, Nationaal Initiatief Hersenen en Cognitie NIHC, under the Partnership program Healthy Lifestyle Solutions (grant no. 12014). A.V.H. is a paid member of Philips Research.
Orr et al., 2015, Canada, [36]	Guy Faulkner is supported by the Canadian Institutes of Health Research-Public Health Agency of Canada (CIHR-PHAC) Chair in Applied Public Health program.
Powierza et al., 2017, USA, [37]	This work was supported by National Institutes of Health grants F30NS090816, T35DK007386, and T32ATT003378 and internal funding from the Matthew Gfeller Sport-Related Traumatic Brain Injury Research Center.
Vooijs et al., 2014, The Netherlands, [38]	None declared.
Boeselt et al., 2016, Germany, [39]	The authors have no support or funding to report.
Ari Wibowo et al., 2020, Indonesia, [40]	None declared.
Arrogi et al., 2018, Belgium, [41]	None declared.
Bort-Roig et al., 2020, Spain, [42]	This study was funded by the Carlos III Health Institute (W@Wapp-Diab; PI17/01788).
Ehrlich et al., 2021, USA, [43]	Dr. Ehrlich is supported by grant K01 DK105106 from the National Institute of Diabetes and Digestive and Kidney Diseases.
Gill et al., 2018, UK, [44]	The EuroFIT study [see http://eurofitfp7.eu] is funded by the European Union's Seventh Framework Program (FP7) for research technological development and demonstration under Grant Agreement no: 602170. PAL Technologies Ltd is manufacturer of the activPAL and SitFIT and is a partner in EuroFIT. Douglas Maxwell, Nikos Mourselas and David Loudon work for PAL Technologies Ltd.
Heyken et al., 2021, Germany, [45]	None declared.

Jansson et al., 2022, Australia, [46]	This work is supported by the National Health and Medical Research Council of Australia (Grant number: APP1134914). MD is supported by a Career Development Fellowship (Grant number: APP1141606) from the National Health and Medical Research Council of Australia. DL is supported by a Senior Research Fellowship (Grant number: APP1154507) from the National Health and Medical Research Council of Australia. https://www.nhmrc.gov.au/. All funders had no role in study design, data collection and analysis, decision to publish, or preparation of the manuscript. There was no additional external funding received for this study.
Karinharju et al., 2021, Australia and Finland (50/50), [47]	This work was supported by 1) The University of Queensland, Human movement and nutrition sciences, PhD candidature funding; 2) Motor Accident Insurance Commission Scholarship (Grant number 2159179); and 3) Suomen Kulttuurirahasto (FI)(Grant number 75162323)
Murakami et al., 2019, Japan, [48]	ST reported receiving research funding from Omron Health Care Inc. No other disclosures were reported.
Toledo et al., 2017, USA, [49]	This study was funded in part by the Virginia G. Piper Charitable Trust. This research was partially supported by resources and the use of facilities at the Phoenix Veterans Affairs Health Care System.
Zhuo et al., 2021, Canada, [50]	None declared.
**Studies evaluating self-assessment tools assessing nutritional intake (*n* = 7)**	
Fukuo et al., 2009, Japan, [51]	This study was partly funded by SHARP Corporation, Osaka, Japan (K.Y.).
Fuller et al., 2017, Australia, [52]	None declared.
Goodman et al., 2015, Canada, [53]	Funding was provided by a Canadian Institutes for Health Research Frederick Banting Doctoral Scholarship (grant #596230) awarded to SG. Naveen Agarwal, PhD, is acknowledged for development and ownership of the Vitamin D Calculator app. Scott Taylor (ShuffleCloud Solutions) is acknowledged for technical work on the Vitamin D Calculator app and database support. Wray Hutton is acknowledged for technical support with the University of Guelph database. Sonya Nakoneczny is acknowledged for contributions as a research assistant. There are no conflicts of interest to disclose.
Hutchesson et al., 2013, Australia, [54]	Funding for this study was received from an Australian Post-graduate Award scholarship, and a scholarship top-up from SP Health Co. Pty Ltd (MJH), as well as a NHMRC Career Development Award Fellowship (CEC).
McClung et al., 2009, USA, [55]	This research was funded by Medical Research Materiel Commend, United States Army.
Matsuzaki et al., 2017, Japan, [56]	No funding was received for this research.
Ocké et al., 2021, The Netherlands, [57]	This research was funded by the Dutch Ministry of Health, Welfare, and Sports.
**Studies evaluating self-assessment tools assessing psychological stress (*n* = 1)**	
Þórarinsdóttir et al., 2019, Denmark, [58]	The study was funded by the Lundbeck foundation and the AP Møller foundation for Icelandic students in Denmark. The funders had no role in the study design, data collection, analyses, and preparation of the manuscript.
**Studies evaluating self-assessment tools assessing multiple lifestyle domains (*n* = 1)**	
Swendeman et al., 2018, USA, [59]	This work was sponsored by NHLBI Grant 5RC1HL099556 to the last author (PI) and the co-authors as co-investigators. Comulada's time was also supported by NIMH Grant K01MH089270. Swendeman's time was supported by a scholar award from the William T Grant Foundation (#180039). Other support was provided by: the Center of HIV Identification, Prevention, and Treatment (CHIPTS) NIMH Grant MH58107; the UCLA Center for AIDS Research (CFAR) Grant 5P30AI028697; and the National Center for Advancing Translational Sciences through UCLA CSTI Grant UL1TR000124. The content is solely the responsibility of the authors and does not necessarily represent the views of the NIH.
