# Supplementary material for: Validity of self-assessment tools for cardiovascular risk behaviors: A systematic review
Source: Am J Prev Cardiol. 2025 Oct 7;24:101316. doi: 10.1016/j.ajpc.2025.101316 (PMC12663659; doi:10.1016/j.ajpc.2025.101316)
Supplement: Supplementary file 5 [file mmc5.pdf]

# Appendix E – QUADAS-2 risk of bias assessment for each study

| Study details | Risk of bias | | | | Applicability concerns | | |
|---|---|---|---|---|---|---|---|
| | **Patient selection** | **Index test** | **Reference standard** | **Flow and timing** | **Patient selection** | **Index test** | **Reference standard** |
| **Studies evaluating self-assessment tools assessing physical activity (*n* = 22)** | | | | | | | |
| Åkerberg et al., 2016, Sweden, [30] | ✓ | ✓ | ✓ | ✓ | ✓ | ✓ | ✓ |
| Chowdhury et al., 2017, UK, [5] | ✓ | ✓ | ✓ | ✓ | ✓ | ✓ | ✓ |
| Dooley et al., 2017, USA, [31] | ✓ | ✓ | ✓ | ✓ | ✓ | ✓ | ✓ |
| Gomersall et al., 2016, Australia, [32] | ✓ | ✓ | ✓ | ✓ | ✓ | ✓ | ✓ |
| Holbrook et al., 2009, USA, [33] | ✗ | ✓ | ✓ | ✗ | ✓ | ✓ | ✓ |
| Leth et al., 2017, Denmark, [34] | ? | ✓ | ✓ | ✓ | ✓ | ✓ | ✓ |
| Middelweerd et al., 2017, The Netherlands, [35] | ✓ | ✓ | ✓ | ✓ | ✓ | ✓ | ✓ |
| Orr et al., 2015, Canada, [36] | ✓ | ✓ | ✓ | ✓ | ✓ | ✓ | ✓ |
| Powierza et al., 2017, USA, [37] | ✓ | ? | ✓ | ✓ | ✓ | ✓ | ✓ |
| Vooijs et al., 2014, The Netherlands, [38] | ? | ✓ | ✓ | ✗ | ✓ | ✓ | ✓ |
| Boeselt et al., 2016, Germany, [39] | ✓ | ✓ | ✓ | ✓ | ✓ | ✓ | ✓ |
| Ari Wibowo et al., 2020, Indonesia, [40] | ? | ✓ | ✓ | ✓ | ? | ✓ | ✓ |
| Arrogi et al., 2018, Belgium, [41] | ✓ | ✓ | ✓ | ✓ | ✓ | ✓ | ✓ |
| Bort-Roig et al., 2020, Spain, [42] | ✓ | ? | ✓ | ✓ | ✓ | ? | ✓ |
| Ehrlich et al., 2021, USA, [43] | ✓ | ✓ | ✗ | ✓ | ✓ | ✓ | ✗ |
| Gill et al., 2018, UK, [44] | ? | ✓ | ✓ | ✓ | ✓ | ✓ | ✓ |
| Heyken et al., 2021, Germany, [45] | ✗ | ✗ | ✓ | ✗ | ✗ | ✗ | ✓ |
| Jansson et al., 2022, | ✓ | ✓ | ✗ | ✓ | ✓ | ✓ | ✗ |

| | | | | | | | |
|---|---|---|---|---|---|---|---|
| Australia, [46] | | | | | | | |
| Karinharju et al., 2021, Australia and Finland (50/50), [47] | ✓ | ✓ | ✗ | ✓ | ✓ | ✓ | ✗ |
| Murakami et al., 2019, Japan, [48] | ✓ | ✓ | ✓ | ✓ | ✓ | ✓ | ✓ |
| Toledo et al., 2017, USA, [49] | ✓ | ✓ | ✓ | ✓ | ✓ | ✓ | ✓ |
| Zhuo et al., 2021, Canada, [50] | ✗ | ✓ | ✓ | ✓ | ✗ | ✓ | ✓ |
| **Studies evaluating self-assessment tools assessing nutritional intake (*n* = 7)** | | | | | | | |
| Fukuo et al., 2009, Japan, [51] | ✓ | ✓ | ✓ | ✓ | ✓ | ✓ | ✓ |
| Fuller et al., 2017, Australia, [52] | ✓ | ✓ | ✓ | ✓ | ✓ | ✓ | ✓ |
| Goodman et al., 2015, Canada, [53] | ? | ? | ? | ✓ | ✓ | ✓ | ✓ |
| Hutchesson et al., 2013, Australia, [54] | ✓ | ✓ | ✓ | ✓ | ✓ | ✓ | ✓ |
| McClung et al., 2009, USA, [55] | ✓ | ✓ | ✓ | ✓ | ? | ✓ | ✓ |
| Matsuzaki et al., 2017, Japan, [56] | ✓ | ✓ | ✗ | ✓ | ✓ | ✓ | ✓ |
| Ocké et al., 2021, The Netherlands, [57] | ✓ | ✓ | ✓ | ✓ | ✓ | ✓ | ✓ |
| **Studies evaluating self-assessment tools assessing psychological stress (*n* = 1)** | | | | | | | |
| Þórarinsdóttir et al., 2019, Denmark, [58] | ✓ | ✗ | ✗ | ✓ | ✓ | ✗ | ✗ |
| **Studies evaluating self-assessment tools assessing multiple lifestyle domains (*n* = 1)** | | | | | | | |
| Swendeman et al., 2018, USA, [59] | ✓ | ✓ | ✓ | ✓ | ✓ | ✓ | ✓ |

✓ indicates low risk; ✗ indicates high risk; ? indicates unclear risk.
